# North American pitseed goosefoot (*Chenopodium berlandieri*) is a genetic resource to improve Andean quinoa (*C. quinoa*)

**DOI:** 10.1038/s41598-024-63106-8

**Published:** 2024-05-29

**Authors:** Peter J. Maughan, David E. Jarvis, Eulogio de la Cruz-Torres, Kate E. Jaggi, Heather C. Warner, Ashley K. Marcheschi, H. Daniel Bertero, Luz Gomez-Pando, Francisco Fuentes, Mayela E. Mayta-Anco, Ramiro Curti, Elodie Rey, Mark Tester, Eric N. Jellen

**Affiliations:** 1 Brigham Young University, Provo, UT USA; 2 Instituto Nacional de Investigaciones Nucleares, Ocoyoacac, Mexico; 3 Universidad de Buenos Aires, Buenos Aires, Argentina; 4 Universidad Nacional Agraria La Molina, La Molina, Peru; 5 Universidad Pontificia Católica de Chile, Santiago, Chile; 6 Universidad Nacional de San Agustín, Arequipa, Peru; 7 Universidad Nacional de Salta, Salta, Argentina; 8 King Abdullah University of Science & Technology, Thuwal, Saudi Arabia

**Keywords:** Agricultural genetics, Genomics

## Abstract

Pitseed goosefoot (*Chenopodium berlandieri*) is a free-living North American member of an allotetraploid complex that includes the Andean pseudocereal quinoa (*C. quinoa*). Like quinoa, pitseed goosefoot was domesticated, possibly independently, in eastern North America (subsp. *jonesianum*) and Mesoamerica (subsp. *nuttaliae*). To test the utility of *C. berlandieri* as a resource for quinoa breeding, we produced the whole-genome DNA sequence of PI 433,231, a huauzontle from Puebla, México. The 1.295 Gb genome was assembled into 18 pseudomolecules and annotated using RNAseq data from multiple tissues. Alignment with the v.2.0 genome of Chilean-origin *C. quinoa* cv. ‘QQ74’ revealed several inversions and a 4A-6B reciprocal translocation. Despite these rearrangements, some quinoa x pitseed goosefoot crosses produce highly fertile hybrids with faithful recombination, as evidenced by a high-density SNP linkage map constructed from a Bolivian quinoa ‘Real-1’ × BYU 937 (Texas coastal pitseed goosefoot) F_2_ population. Recombination in that cross was comparable to a ‘Real-1’ × BYU 1101 (Argentine *C. hircinum*) F_2_ population. Furthermore, SNP-based phylogenetic and population structure analyses of 90 accessions supported the hypothesis of multiple independent domestications and descent from a common 4 × ancestor, with a likely North American Center of Origin.

## Introduction

The pseudocereals represent a group of mostly underutilized crops with tremendous potential for diversifying worldwide food production and reducing protein malnutrition. One of the most versatile of these crops is quinoa (*Chenopodium quinoa* Willd.), an increasingly popular Andean-origin plant possessing excellent seed protein for human nutrition, and which is well adapted in temperate drought- and salinity-stressed environments. Quinoa is one of the domesticated members of the Allotetraploid Goosefoot Complex (ATGC; 2*n* = 4*x* = 36, AABB genome)^1–3^. The other ATGC taxa are wild-weedy North American *C. berlandieri* Moq.; wild-weedy South American *C. hircinum* Schreb.; and the domesticated Mesoamerican seed/vegetable *C. berlandieri* subsp. *nuttaliae*^4^. There is archaeological evidence that quinoa and its companion weed, *C. quinoa* var. *melanospermum* (*quinua negra*, *ayara,* or *ajara*), were domesticated from a wild tetraploid taxon, possibly *C. hircinum*, earlier than 3,500 ybp in the central Andean Highlands^5–8^ – the region where the majority of commercial quinoa is still produced. Quinoa is hypothesized to have eventually spread to the coastal region of Araucania in South-Central Chile, which is the source of much of the germplasm currently being grown and bred for commercial production in low-altitude environments around the world. Although not taxonomically distinguished from highland Andean quinoa, the lowland Chilean-origin *quingua* or *zawe* variety ‘QQ74’ that was sequenced as a quinoa reference^9^ is now known to harbor a chromosome 3B pericentric inversion that is functionally large enough to constitute a subspecies-defining rearrangement^10^.

North American wild *C. berlandieri* is commonly known as pitseed goosefoot and inhabits disturbed environments on well-drained soils throughout North America. The Flora of North America treatment by Clemants and Mosyakin (http://www.efloras.org/florataxon.aspx?flora_id=1&taxon_id=106630) describes six free-living botanical varieties of the species^3^. The berlandieri and boscianum botanical varieties are native to the southeastern United States, with the former restricted to the South Texas Plains and the latter inhabiting beaches, estuaries, and saline bayous along the Gulf of Mexico. Both ecotypes usually have a fishy odor, similar to diploids *C. vulvaria* and *C. watsonii* and AABB *C. hircinum*, due to the accumulation of trimethylamine (TMA), a secondary metabolite formed from the incomplete breakdown of the stress-related molecules choline and betaine^11^. Variety bushianum has large (up to 2 mm diameter) seeds and is a weed of river bottoms and agricultural fields in eastern North America. The macrocalycium botanical variety is a bushy plant that grows along the high-tide line where seaweed accumulates on New England beaches. Sinuatum is an ecotype native to southwestern North America, with prominent basal and elongated terminal lobes and lacking yellow-orange pigmentation at the stylar attachment point on the pericarp. Zschackei is described as a catch-all variety, encompassing strains of pitseed goosefoot that do not fit into the other classifications.

Where cultigens are grown in sympatric environments with wild ATGC members they are subject to exoferalization^12,13^ thereby forming crop-weed complexes through outcrossing^14^. Within Mesoamerica *C. berlandieri* subsp. *nuttaliae* exists in three to four domesticated to wild forms that are identifiable by the thickness of the testa^2^: as black-seeded, leafy vegetable *quelites*; as an immature panicle vegetable, *huauzontle*; as a specialty seed crop in Michoacan known as *chia roja*^4^; and possibly as a distinct semi-weedy form, sometimes known as pueblense, though originally described as the separate taxon *C. pueblense*^15^. A similar situation exists in the Andes, where quinoa is cultivated alongside semi-weedy *C. quinoa* subsp. *melanosperma*, subsp. *milleanum*, and in Chile and Argentina in close proximity to weedy *C. hircinum*^6,7^. As in Mesoamerica, there are multiple domesticated to semi-domesticated eastern North American forms of pitseed goosefoot in the archaeological record, among them a small-seeded, thick-exoderm (testa) weedy form most likely consumed as a leafy vegetable; a thin-testa form, known as subsp. *jonesianum*, abundant in sites throughout eastern North America (ENA) as far north as Ontario in Canada dating from ~6000 ybp^16^ ; and a third ENA form, similar to *huauzontle* seeds in that they lack the pigmented exoderm^17^. Unlike the situation in Mesoamerica, only the wild-weedy form was still extant in ENA at the time of Anglo-French colonization of North America^18^. The overriding question for archaeology is whether the known ATGC domesticates arose independently in the Andean Highlands, the South-Central Chilean Littoral, Mesoamerica, and ENA. The alternative hypothesis is that they all trace back to a single domestication event, most likely as *C. quinoa* in the Andes^1^. Wilson and Heiser hypothesized that the Andean and Mesoamerican taxa were independently domesticated, based on isozyme and morphological comparative analyses^2^.

The salient evolutionary question is whether the ATGC members are monophyletic or, alternatively, if they arose via separate allopolyploidization events. If the latter hypothesis is correct, they either arose from crosses between the same ancestral diploids or from one or more different parents. To date, genome sequence-, single gene-, and DNA marker-based comparisons of the complete quinoa^9,10^ and fragmentary wild pitseed goosefoot^19^ genomes have confirmed their synteny, extensive collinearity, and common AABB genome composition, indicative of a monophyletic origin^20–22^.

Additionally, the increasing worldwide demand for quinoa as a human staple food justifies an in-depth investigation of huauzontle and warm-temperate pitseed goosefoot as potential germplasm resources for quinoa breeding^3^. This is especially important as quinoa breeding programs have emerged in warm, lowland production environments in Sub-Saharan Africa^23,24^, North Africa^25–27^, Southeast Asia^28^, South Asia^29,30^, lowland South America^31,32^, and North America^33,34^. Since pitseed goosefoot is found primarily in the United States, a country with a policy of open exchange of genetic resources, this species could provide unrestricted lowland germplasm for international quinoa improvement^3^.

To more precisely answer these archeological and evolutionary questions, and to determine the potential breeding value of pitseed goosefoot for quinoa and huauzontle improvement, we embarked on a multi-faceted study of pitseed goosefoot, including the following: (i) establishment of a high-quality reference genome sequence for huauzontle and its comparison with quinoa and A- and B-genome diploid references; (ii) comprehensive collection and resequencing of 90 ATGC genotypes, with subsequent high density SNP-genotyping used to elucidate evolutionary relationships within the ATGC; and iii) investigation of the potential of intertaxa cross hybridization (quinoa × pitseed goosefoot, var. bosicanum) for genetic improvement (i.e., are interspecific crosses viable and does recombination occur normally between the homologous chromosomes).

## Methods

### Genome sequence resources

Huauzontle (*C. berlandieri* subsp. *nuttaliae*) PI 433231 was selected as the reference pitseed goosefoot genotype. It originated from Atlixco, Puebla, Mexico (18.895833 N*,* -98.361389 W*,* 1880 masl) and was previously shown to have a *waxy* phenotype, being homozygous for recessive alleles at the *GBSSIa* and *GBSSIb* loci in the A and B sub-genomes, respectively^35^. A single plant of PI 433231 was grown hydroponically in a growth chamber set to a photoperiod of 11 h with broad-spectrum lighting. Temperature controls were set between 18 and 20 °C. The hydroponics solution was made using 27 g of MaxiGrow**®** Hydroponics Plant Food (General Hydroponics, Sebastopol, CA) dissolved in 16 L of deionized water and was replaced every two weeks. Prior to extraction, the plant was dark-treated for 72 h. Young leaf tissue was harvested and high molecular weight DNA was extracted using a CTAB-Qiagen Genomic-tip protocol as described by Vaillancourt and Buell^36^. A SageElf (Sage Science, Inc., Beverly, MA, USA) was used to produce large-insert SMRTBell libraries (> 20 kb) that were sequenced using P6-C4 chemistry on a Sequel II instrument to produce CLR data (Pacific BioSciences, Menlo Park, CA) at the BYU DNA Sequencing Center (Provo, UT).

For whole-genome polishing, DNA from the sequenced plant was sent to Novogene (San Diego, CA) for Illumina short-read (2 × 150 bp) sequencing from standard 500‐bp insert libraries. Trimmomatic^37^ v0.35 was used to remove adapter sequences and leading and trailing bases with a quality score below 20 or average per-base quality of 20 over a four-nucleotide sliding window. After trimming, any reads shorter than 75 nucleotides in length were removed. For contig scaffolding, leaf tissue from the huauzontle plant was sent to Phase Genomics (Seattle, WA) for Hi-C library construction and paired-end sequencing (100 M read pairs / Gb; 2 × 150 bp). For genome annotation, total RNA was extracted separately from a single plant (PI 433,231) for each tissue type including panicles, leaves, stems at the 8-week growth stage, and 7-day old seedlings (grown on filter paper), using a Qiagen RNeasy Plant mini kit (Germantown, MD) following the manufacturer’s suggested protocol. The quality and quantity of the RNA from each tissue was assessed using an Agilent Bioanalyzer (Santa Clara, CA). RNA from each tissue type was pooled in equal molar ratios to create a single bulk sample which was subsequently sequenced using the PacBio (Menlo Park, CA) Iso-Seq platform on a Sequel II instrument (BYU DNA Sequencing Center, Provo, UT). The cDNA was prepared from the bulk sample using a NEBNext® single cell/low input cDNA synthesis and amplification kit (E6421L; New England BioLabs, Ipswich, MA). IsoSeq libraries were prepared using the SMRTbell v3.0 library prep kit (Menlo Park, CA). The RNAseq data were trimmed using Trimmomatic as described previously and aligned to the final assembly using HiSat2^38^ v2.1 with default parameters and maximum intron length set to 50,000 bp. The resulting SAM file was sorted and indexed using SAMtools^39^ v1.6 and assembled into putative transcripts using StringTie^40^ v1.3.4.

### Genome assembly

Canu^41^ v1.9, with default parameters, was used to construct the primary contig assembly of huauzontle. The primary assembly was polished twice with Illumina data using Arrow from the GenomicConsensus package in the Pacific BioSciences SMRT portal v5.1.0, followed by a single round of insertion/deletion correction using PILON^42^ v0.22. The polished genome was then scaffolded to chromosome scale using the Hi-C data. Hi-C reads were aligned to the primary contig assembly using the Burrows-Wheeler Aligner (BWA)^43^. Only paired-end reads that uniquely aligned to contigs were retained for downstream analyses. Contigs were clustered, ordered, and oriented using Proximo™, an adapted proximity-guided assembly platform based on the LACHESIS method^44,45^ with proprietary parameters developed at Phase Genomics^46^. Gaps between scaffolds within the assembly were filled with 100 Ns. To assemble the chloroplast, the *C. quinoa* chloroplast genome (GenBank acc: MK159176) was used as a custom seed library for GetOrganelle^47^ v.1.7.7 using default parameters for plant plastomes (embplant_pt). The chloroplast assembly was annotated using GeSeq^48^.

### Genome annotation, repeat analysis and validation

Species-specific repeats were first identified by RepeatModeler^49^ v1.0.11. RepeatMasker^50^ v4.0.9 was then used to identify and classify repeat elements within the assembled genome relative to the Repbase-derived RepeatMasker libraries (Dfam^51^ 3.0; 20,190,227). Repeats were annotated for each sub-genome separately and visualized as Kimura divergence repeat landscapes (adjusted for CpG sites and transformed based on the total genome size) using the calcDivergenceFromAlign.pl scripts, which are provided with RepeatMasker. To estimate a rough time scale for the divergence, we used the equation: time = divergence/2r, where r = 1.3 × 10^–8^ mutations per site per year, which is the previously estimated rate of LTR element sequence evolution in rice^52^.

The MAKER3^53^ pipeline was used to annotate the genome. Primary evidence files used for the annotation included the species-specific *C. berlandieri* transcriptome (see above), 44,776 *C. quinoa* predicted gene models and their translated protein sequences^9^ as well as all proteins in the highly curated Uniprot Swiss-Prot database (*n* = 561,176; accessed Feb 25, 2019). Ab initio gene prediction was based on *C. berlandieri* specific and *A. thaliana* hidden Markov models for Augustus and SNAP*,* respectively. AED scores, used to assess the quality of the gene predictions, were generated for each of the annotated genes. Putative gene function was identified using BLAST searches of the predicted peptide sequences against the Swiss-Prot database using MAKER’s default cut-off values (1e^–6^). Gene models were also classified as high-confidence and low-confidence based on predicted proteins using custom Python scripts (Supplemental Figure S1). High-confidence models exhibited (1) a BLASTp hit (< 1.0E-10) against the Magnoliopsida TrEMBL^54^ database; (2) a query coverage and length within 25% of the subject coverage and length; and (3) > 66% percent identity between the query and subject. Genome assembly and annotation completeness were assessed using BUSCO^55^ v5.0 with the “long” argument which applies Augustus^56^ optimization for self-training against the Embryophyta (odb10) orthologs database (Supplemental Figures S2, S3).

## Genome structure visualization

The 18-24 J and telomeric repeat regions in the *C. berlandieri* genome were identified using BLAST, as previously described^9^. To locate centromeres, a triplicated centromeric sequence from *C. vulvaria* (GACTTTCATTTGATTCAATTAGCTTTGTTTGAAT) was used as a BLAST query against *C. berlandieri*, and all hits with an ID >  = 95% were kept. For visual purposes, the centromere plot height was capped at 9. Circa (http://omgenomics.com/circa) was used to view the densities of the genes, telomeric repeats, centromeric repeats, and 18-24 J repeats in 500-kb windows for all *C. berlandieri* chromosomes. For SNP markers mapped in the Real-1 × BYU 937 and Real-1 × BYU 1101 populations, Circa was used to plot the genetic distance between mapped markers based on their physical locations in the genome.

Homologous genes within the *C. berlandieri* genome and between the *C. berlandieri* and quinoa (CoGe id65146) genomes were identified using CoGe SynMap^57^. Blocks of syntenic genes were identified with MCScanX^58^ and visualized as dotplots using SynVisio^59^. Homologous genes between *C. watsonii*^60^ and the A subgenomes of *C. berlandieri* and quinoa, and between the B subgenome of *C. formosanum*^61^ and the B subgenomes of *C. berlandieri* and quinoa, were identified using BLASTp with the num_descriptions 5, -num_alignments 5, and -evalue 1e-10 settings applied. Blocks of syntenic genes were identified with MCScanX and visualized as treeview plots with SynVisio.

### ATGC resequencing panel and SNP calling

A resequencing panel consisting of 90 ATGC genotypes (Supplemental Table S1) included 16 genotypes of *C. quinoa*; five of *C. hircinum*; 17 strains of *C. berlandieri* subsp. *nuttaliae*; and 52 accessions of *C. berlandieri* subsp. *berlandieri*. Seeds were germinated and seedlings raised according to previously described protocols^60^ and DNA from young leaf tissue was extracted using a standard mini-salts method^62^. DNA was sequenced using Illumina (2 × 150 bp) to reach 10X coverage by Novogene, Inc. (San Diego, CA) ^36^. Reads were trimmed using Trimmomatic as described previously and then mapped to the final *C. berlandieri* assembly using Minimap2^63^ v2.1 with the “sr” preset. The resulting BAM files were sorted, followed by duplicate reads and quality alignments (< Q45) removal using the subtools, “sort”, “markdup”, and “view” of SAMtools^39^ v1.6, respectively. Single-nucleotide polymorphisms (SNPs) were called from the BAM files using InterSNP, a program within the BamBam^64^ v1.4 pipeline which produced a SimpleSNP file containing the SNP and genomic location for each of the accessions relative to the *C. berlandieri* genome. InterSNP parameters included a minimum read coverage of two, and since these species are primarily autogamous, heterozygous SNPs were excluded from downstream analyses.

### Phylogenetic and STRUCTURE analyses

SNPhylo^65^ v20160204, which reduces overweighting of SNPs, was used to further filter the SNP data set using a 500,000 bp sliding window with a linkage disequilibrium threshold of 0.3. SNPs with missing data or a minor allele frequency < 0.15 were removed from the final dataset. SNPphylo results were then analyzed with IQ-TREE^66^ to generate a phylogenetic tree based on the maximum likelihood estimation using a general time reversible substitution model, including a gamma distribution for rate heterogeneity among sites with a correction applied to account for ascertainment bias (GTR + G + ASC). Bootstrap nearest neighbor interchange (-bnni) was used to improve the accuracy of the bootstrap analysis (*n* = 1000). Trees were visualized with FigTree v1.4.4^67^. Population structure was evaluated using STRUCTURE v2.3.4^68^ with a range of K = 1 through K = 10 with 20,000 MCMC reps after an initial burn-in period of 10,000 with the assumptions of no admixture and correlated allele frequencies. StructureSelector^69^ was used to identify the optimal number of clusters (K) using the Puechmaille method^70^. CLUMPAK^71^ and STRUCTURE PLOT V2.0^72^ were used to draw STRUCTURE bar plots sorted by individual Q matrices.

### Linkage map construction

Two F_2_-based linkage maps were generated. The first map was from a cross between the *C. quinoa* variety ‘Real-1’ (maternal parent) and the *C. berlandieri* var. *boscianum* accession ‘BYU 937’. ‘Real-1’ was obtained from A. Bonifacio at the Foundation for Promotion and Investigation of Andean Products (PROPINA), La Paz, Bolivia, while ‘BYU 937’ was derived from a single plant collected in July of 2009 along the shoreline of Galveston Bay in Texas City, TX. The F_1_ plant bore black-testa seeds of intermediate size **(**~ 1.5 mm) and smelled strongly of trimethylamine (TMA) like its ‘BYU 937’ male parent, indicative of a successful cross hybridization. The second linkage map was generated from a cross between the *C. quinoa* accession ‘Real-1’ (maternal parent) and a *C. hircinum* plant ‘BYU 1101’ collected by D. Bertero in Ceres, Argentina that was previously described and genotyped by Jarvis et al.^9^ Seeds from F_**1**_ and F_**2**_ plants from both populations were scarified by nicking the testa with a scalpel and germinated in Petri plates on filter paper saturated with 100 ppm GA_3_ and 30 mM KNO_3_ at ~ 20º C. DNA was extracted from young leaves of F_2_ plants using a standard mini-salts method^62^. The F_2_ population was genotyped by sequencing (GBS) using the restriction endonucleases *Bfa*I and *Nsi*1 at the University of Wisconsin Biotechnology Center (Madison, WI). GbS FASTQ files were first cleaned, trimmed, and filtered using the Fastp^73^ v0.20.1. Stacks^74^ v2.55 was used to demultiplex the sequencing data, followed by mapping of the reads to the *C. quinoa* reference v.2.0^10^ and the de novo PI 433321 huauzontle assembly using Bowtie2^75^ v.2.3 with “local” and “very-sensitive-local” elicited. The subprograms gStacks and Populations were then used to make genotypic calls and produce map files for JoinMap^76^ v5.0. The mapping files were manually filtered to remove individual samples with > 30% missing data, or segregation distortion > 1e-8 relative to the expected segregation ratio for an F_2_ population (1:2:1)—leaving 154 and 86 F_2_ plants after filtering in the Real-1 × BYU 937 and Real-1 × BYU 1101 populations, respectively. Genetic linkage maps were prepared from segregating, high-confidence SNPs that were grouped based on the independence logarithm of odds (LOD) scores (≥ 5.0) using the G2 statistic followed by ordering within groups using regression mapping. After the addition of each SNP locus to the map, a ripple test was conducted within the JoinMap software^76^ for all order permutations across a moving three-SNP window to test for goodness-of-fit and to assure the optimal map order. Tandem Repeat Annotation and Structural Hierarchy^77^ v1.2 (TRASH) was employed to identify and plot the location of the pericentric inversion on chromosome 3B in the quinoa reference genome (Supplemental Figure S4).

### Extranuclear inheritance

To determine the inheritance pattern for the extranuclear genomes, Illumina short reads (2 × 150 bp) for the parents of the mapping populations (Real-1, BYU 937 and BYU 1101) as well as their F_1_ hybrids were aligned against the reference chloroplast and mitochondria^78^ (MK159176 and MK182703, respectively) genomes using minimap2 and called for SNPs using InterSNP as described previously, except that the InterSNP parameters included a minimum read coverage of six. Since the chloroplast is known to contain two large, inverted repeats, these regions were removed prior to the analysis (Supplemental Figure S5).

## Results

### Whole-genome assembly and annotation of huauzontle PI 433,231

**Figure 1:**
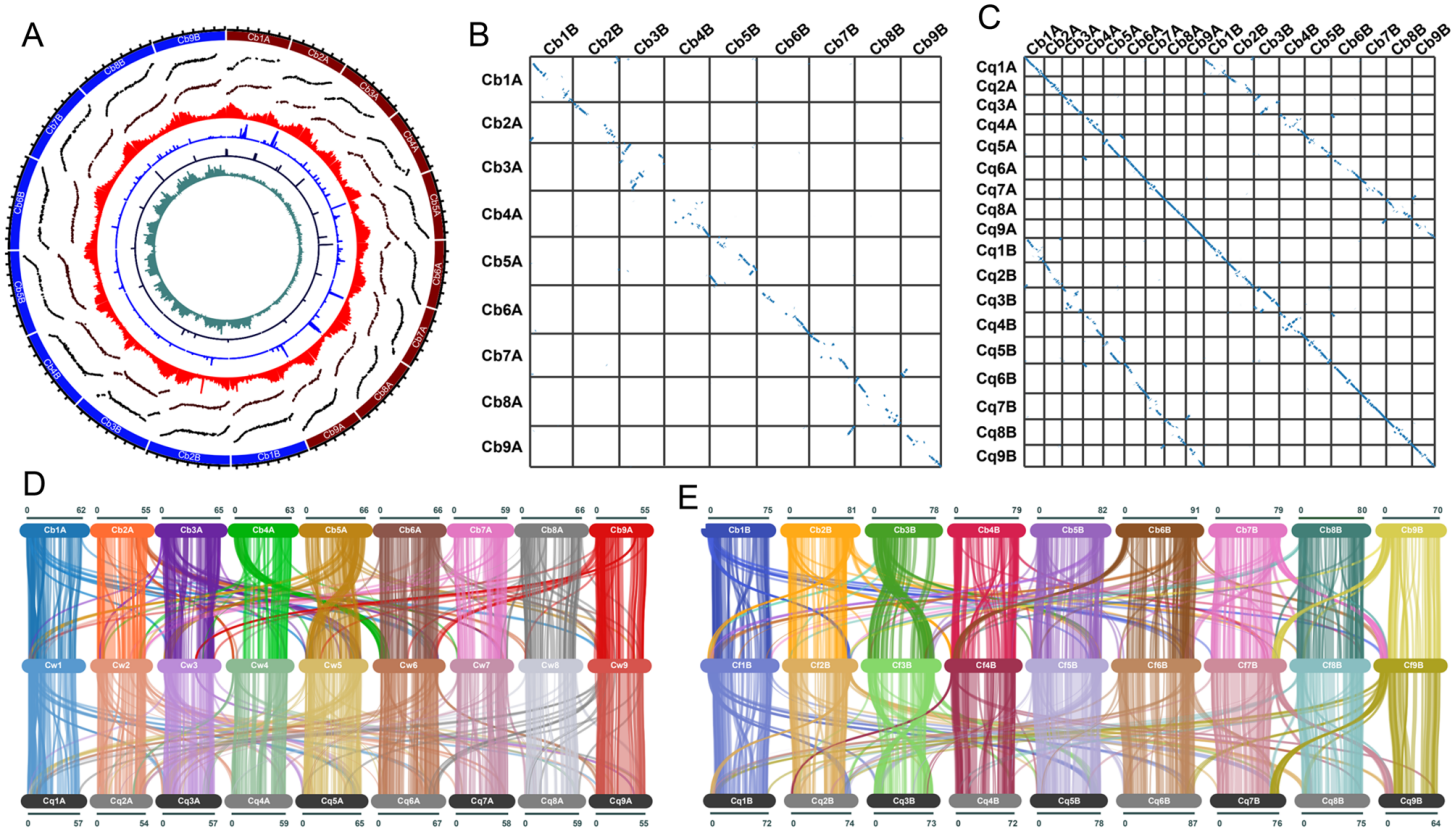
Genome structure and synteny of *C. berlandieri*. (**A**) Overview of the structure and composition of huauzontle chromosomes in 500-kb windows. From outside to inside, tracks represent chromosome names and sizes (tick marks represent 10 Mb intervals); genetic distance (*y*-axis) between markers mapped in a Real-1 × BYU 937 population as a function of their physical position on the huauzontle chromosomes; genetic distance (*y*-axis) between markers mapped in a Real-1 × BYU 1101 population as a function of their physical position on the huauzontle chromosomes; annotated gene density; centromeric repeat density; telomeric sub-repeat density; and 18-24 J repeat density. (**B**) Dotplot of the positions of homologous gene pairs between the A and B subgenomes of huauzontle. (**C**) Dotplot of the positions of homologous gene pairs between the huauzontle and quinoa genomes. (**D**) Synteny plot connecting the positions of homologous gene pairs between *C. watsonii* (middle) and the A subgenomes of huauzontle (top) and quinoa (bottom). (**E**) Synteny plot connecting the positions of homologous gene pairs between the B subgenome of *C. formosanum* (middle) and the B subgenomes of huauzontle (top) and quinoa (bottom).

The primary contig genome assembly of huauzontle was generated using Canu^41^ from 78.1 Gb (~ 60X coverage) of sequence data from 5,560,207 reads with a read length N50 of 24,338 bp and a mean read length of 14,320 bp. After polishing, the primary contig assembly had an N50 and L50 of 5.2 Mb and 73, respectively, and consisted of 1,170 contigs (Supplemental Table S2). To further improve the assembly, contigs were scaffolded using chromatin-contact (Hi-C) maps to produce a 1.296 Gb genome assembled into 339 scaffolds with scaffold N50 and L50 values of 70.1 Mb and 9, respectively. The 18 largest scaffolds, corresponding to the 18 haploid chromosomes, comprised 98.2% of the total assembly length, with the longest scaffold spanning 91.0 Mb. The chromosome-scale scaffolds were largely complete, with consensus telomeric repeat sequences detected on at least one end of all chromosomes but one (Fig. 1A). Centromeric repeat sequences were most prominent in the middle of the chromosomes and gene densities, as expected, were highest toward the distal ends (Fig. 1A). Nine pairs of homoeologous chromosomes were identified (Fig. 1B), corresponding to the nine chromosomes from each of the two sub-genomes. Chromosomes in each pair were assigned to the A or B sub-genomes by plotting the frequency of the 18-24 J satellite repeat sequence, which was previously shown to be overrepresented in the B sub-genome^61,79^ . Nine chromosomes, one from each pair, showed a clear overabundance of the 18-24 J repeat (Fig. 1A); these chromosomes were assigned to the B sub-genome with the others assigned to sub-genome A. The chromosomes were numbered 1–9 based on homology to chromosomes from quinoa, which further supported the assignment of chromosomes to the two sub-genomes, as the chromosomes assigned to the A and B sub-genomes in *C. berlandieri* demonstrated the highest degree of synteny with chromosomes from the A and B sub-genomes of quinoa, respectively (Fig. 1C). Analysis of gene synteny between huauzontle, quinoa (QQ74), the A-genome diploid *C. watsonii*^60^, and the B-genome diploid *C. suecicum*^9^ indicated a high degree of collinearity among the chromosomes, although several major chromosome rearrangements were evident, including a complex inversion series on chromosome 5A and a reciprocal 4A-6B translocation (Fig. 1D & E).

**Figure 2:**
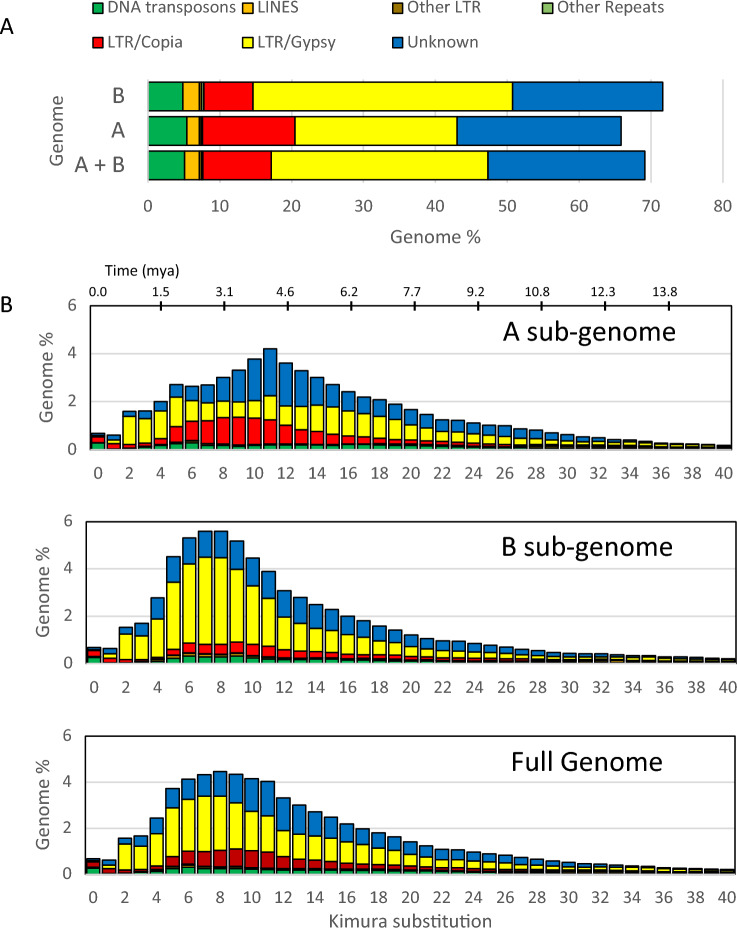
Repeat analysis of *C. berlandieri*. (**A**) Abundance of the major repetitive element categories for the *A* and* B* sub-genomes in *C. berlandieri* ssp. *nuttaliae*. The *x*-axis shows the percent of the genome for each major repeat category. (**B**) Repeat element landscape plots for the *A* and *B* sub-genomes. The *y*-axis shows repeat element abundance as a percentage of the genome. The *x*-axis shows CpG adjusted Kimura sequence divergence and evolutionary time (calculated using LTR sequence evolution rate estimates in rice of 1.3 × 10^–8^ mutations per site per year) relative to consensus sequences for TE superfamilies.

RepeatModeler and RepeatMasker were used to analyze the repeat fraction of the final assembly and to mask the genome prior to annotation. Approximately 64.8% (839 Mb) of the huauzontle genome was annotated as repetitive, which is similar to the repeat fraction classified for other members of the *Chenopodium* genus with reference genomes, including *C. quinoa* (AABB; 4*x* = 36; 64.5%)^9^, *C. formosanum* (BBCCDD; 6*x* = 54; 65.7%)^74^, *C. pallidicaule* (AA; 2*x* = 18; 54.4%)^80^ and *C. watsonii* (AA; 2*x* = 18; 60.4%)^60^*.* The lower than expected repeat fraction in the *C. pallidicaule* assembly was likely due it being a short-read based assembly. The most common repetitive elements identified were long-terminal retrotransposons (LTR-RT), specifically *Gypsy*- (28.1%) and *Copia*-like (8.8%) elements, with 18% of the repetitive elements classified as unknown—reflecting a lack of Chenopodiaceae-specific transposable elements in the Dfam database. The B sub-genome was ~ 28% larger than the A sub-genome (715 vs. 557 Mb, repectively), but despite this increase in sub-genome size the amount of non-repetitive genome sequence was nearly identical between the two sub-genomes (225 and 228 Mb for the A and B sub-genomes, respectively). This suggests that the repetitive fraction accounts for nearly all of the size difference between the two sub-genomes. Indeed the A sub-genome was composed of 59.6% repetitive elements whereas the B sub-genome was composed of 68.1% repeats. *Gypsy*-like and *Copia*-like LTR elements were the most abundant transposons classified in both sub-genomes (Fig. 2A) with *Gypsy* elements showing a marked increase in the B sub-genome at *K* = 7 and *Copia* showing a slight increase in the A sub-genome at *K* = 9 sequence divergence (Fig. 2B). Taking into account the change in abundance of both elements, the increase in *Gypsy* elements in the B sub-genome accounts for an additional 132 Mb of LTR sequence or ~ 98% of the B sub-genome size change, and while relatively small (23 Mb) the increase in abundance of the LTR copia-like element accounts for ~ 89% of the change in size of the A sub-genome. The *Copia* and *Gyspy* elements peak in the A and B sub-genomes at approximately between 3.1–3.8 and 2.3–3.1 MYA, repectively, which roughly corresponds with the estimated origins of the A and B genomes^81^.

Annotation of the huauzontle genome was performed by MAKER^53^ using multiple lines of evidence, including a de novo* C. berlandieri*-specific transcriptome, the gene models from *C. quinoa*^9^, and ab initio models based on Augustus and SNAP. A total of 62,504 mRNA models and 3,423 tRNA genes were identifed, with 40,493 classified as high confidence. The average mRNA and protein spanned 4,098 bp and 366 aa, respectively. A Benchmarking of universal, single-copy orthologs (BUSCO) analysis indicated that the huauzontle genome assembly is largely complete, with 97.7% of BUSCO genes identified as complete relative to Embryophyta datasets, with 80.7% of the conserved orthologs being duplicated as expected for a polyploid species. An analysis of the assembly with Inspector^82^, a reference-free, long-read de novo assembly evaluator, identified an assembly error rate of 28.4 small-scale assembly errors per Mb, of which 81.5%, 6.1% and 12.4% were identified as base substitutions, small-scale (< 50 bp) expansions and small-scale collapses, respectively, resulting in an overall genome quality score of 44—which is slightly better than the QV reported for version 2.0 of quinoa (QV = 37)^10^ and suggestive of a high-quality genome assembly. The annotation contained 1,527 (94.6%) complete COGs, including 357 (22.1%) single-copy and 1,170 (72.5%) duplicated COGs with a total of 42 (2.6%) of the COGs missing (Supplemental Fig. S2). Of the duplicated COGs, 95% were duplicated twice, as would be expected for an allotetraploid species. Nearly all (99%) of the complete COGs were retained in the high confidence annotations. To further assess the quality of the annotations, we analyzed the mean Annotation Edit Distance (AED) which is a specificity and sensitivity measure employed by MAKER. AED values of 0.35 and below are considered high quality annotations^83^. Slightly more than 78% of all gene models had an AED value < 0.35, while 90% of the models in the high confidence set had values < 0.35 (Supplemental Fig. S3).

### Linkage map of Real-1 × BYU 937 and its alignment with quinoa and PI 433,231

To investigate recombination rates in interspecific crosses of *C. quinoa* and *C. berlandieri* (cv. ‘Real-1’ × BYU 937), a linkage map was constructed from 154 F_2_ individuals and 2,473 high confidence SNPs and plotted against the physical genomes of quinoa ‘QQ74’ (Fig. 1A) and huauzontle PI 433231. Linkage analysis identified 18 highly-supported linkage groups (LG), spanning a total distance 2,328 cM, with an average of 137 marker loci per LG. A clear one-to-one correspondence between LGs and the physical chromosomes of the quinoa reference genome was observed (Fig. 1A). Of the 2,473 loci, 1,054 mapped to the A sub-genome, while 1,170 SNPs mapped to the B sub-genome chromosomes of the quinoa reference genome. While the B sub-genome is physically larger than the A subgenome by approximately 26%^10^ (due likely to the expansion of LTRs^73^) the linkage maps are of similar size (1,157 and 1,170 cM), suggesting that repeat expansion in the B subgenome is likely in heterochromatic regions having suppressed recombination and thus do not result in significantly larger linkage groups.

A near-perfect collinear relationship was observed between linkage and physical distance along the sub-genome chromosomes as would be expected for a cross between two genotypes belonging to the same species. As expected, significant suppression of recombination rates at the pericentromeric regions of the chromosomes was observed. This is reflected as substantially reduced linkage distances relative to physical distance (Fig. 1A). It is widely documented that recombination is significantly suppressed in centromeres at rates varying from fivefold to over 200-fold, depending on the species^84^. A large pericentric 3B inversion was detected, which as was previously noted is a structural variant within a subset of Chilean-origin quinoa genotypes, including the ‘QQ74’ reference genome (Supplemental Figure S4)^10^. Both Real-1 and BYU 937 have the ancestral (non-inverted) state of chromosome 3B, thus we expected to see it when comparing the 3B linkage group with the QQ74 reference genome. Since both parents share the same ancestral state, no aberrant chromosome pairing should occur on this chromosome (i.e., normal meiotic pairing of homologous chromosomes should be expected). Indeed, a careful examination of recombination across this region of the 3B chromosome showed that there was ample recombination moving distally from the centromeres, indicating proper pairing of the homologs in the F_1_ hybrid. The linkage map further localized the position of the centromere within the inversion, confirming that the 3B inversion is pericentric (Supplemental Fig. S4A). This was further supported by the mapping of a pericentromeric repeat to a position within the inversion (3B:40–43 Mbp; Supplemental Fig. S4B). Relative to the reference genome, a small terminal inversion was also observed on one end of chromosome 4A. To further investigate this region, a second linkage map was constructed from an interspecific cross of Real-1 × BYU 1101 (Fig. 1A), where BYU 1101 is an accession of the free-living *C. hircinum* from Santa Fe Province in northern Argentina; *C. hircinum* was previously suggested to be the immediate South American ancestor of quinoa^1,4,6,7^. For this population 1,714 high-confidence segregating SNPs were mapped in 86 F_2_ plants. Perhaps surprisingly, in addition to confirming the presence of a small 4A inversion, a larger terminal inversion was identified on chromosome 6A between these two geographically closer parents. This result suggests the 4A inversion is a structural variant of the Real-1 genome, since it was not observed in PI 433231 (Mexican huauzontle), QQ74 (Chilean coastal quinoa), BYU 937 (Texas coastal boscianum), or BYU 1101 (Argentine pampas avian goosefoot).

The Real-1 × BYU 937 and Real-1 × BYU 1101 linkage maps were also plotted against the PI 433,231 pseudomolecule assemblies; poorer alignment of these regression maps in comparison with the QQ74 v.2 assembly suggests structural differentiation between the *C. berlandieri* var. *boscianum* (BYU 937) and *C. berlandieri* subsp. *nuttaliae* (PI 433,231) is substantially greater than BYU 937 and 1101 versus the quinoas (results not shown). The alignments show the T4A-6B interchange, with additional partial-arm displacements on 3B (both maps) and 8B (BYU 1101 map); 5A arm-displacement (both maps); a 5B terminal inversion (both maps); a displaced intercalary segment on 2A (both maps); and possible small telomeric inversions on chromosomes 2B (both maps), 3A (BYU 1101 map), 5B (both maps), and 8B (both maps). Normal recombination distribution patterns were observed along chromosomes 1A, 1B, 4B, 6A, 7A, 7B, 8A, 9A, and 9B in the BYU 1101 map and on these same chromosomes except for a small intercalary 8A inversion in the BYU 937 map.

We also assembled the complete chloroplast genome for *C. berlandieri* subsp. *nuttaliae*, which spanned 152,125 bp (Supplementary Fig. S5A) and investigated the inheritance pattern for the extranuclear genomes in the aforementioned segregating populations. To do this we analyzed the inheritance pattern of 404 polymorphic SNPs located within the chloroplast and mitochondria genomes (Supplemental Fig. S5B) between the parents and their respective F_1_ hybrids. Of the 404 SNPs, including 182 and 222 identified within the chloroplast and mitochondria genomes, respectively, 402 (99.5%) exhibited a maternal inheritance pattern, confirming the uniparental-maternal inheritance of the cytoplasm. These results also confirm that an A-genome diploid was the original female pollinated by a B-genome male in the wide hybridization event that gave rise to the ATGC ancestor.

### Analyses of phylogenetics and diversity in C. berlandieri and other ATGC genotypes

**Figure 3:**
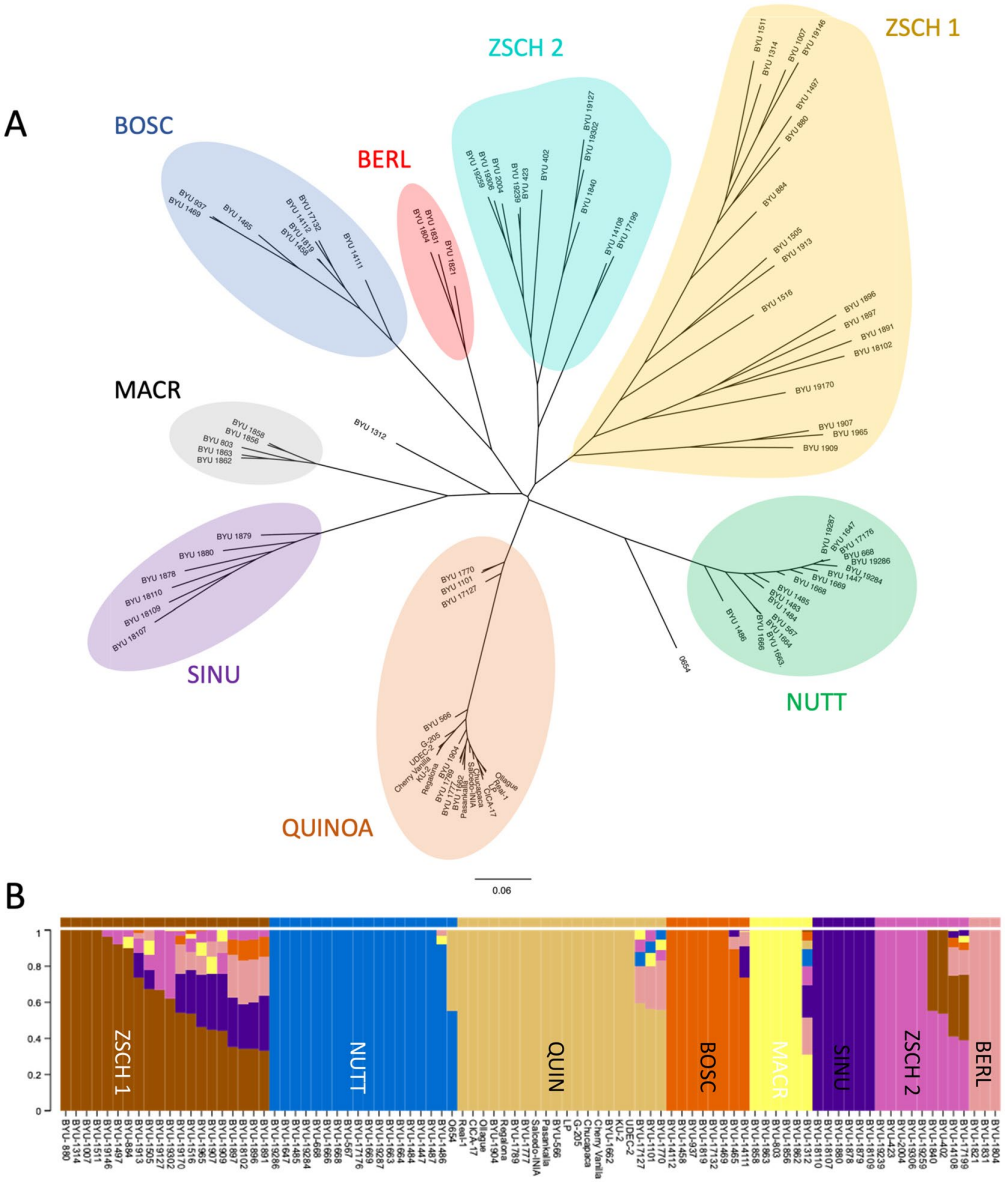
(**A**) Midpoint-rooted tree with colored clades. The maximum likelihood tree was generated using IQ-TREE^66^ and 12,915 SNPs after filtering using the following parameters: < 10% missing data, minor allele frequency < 15%, and linkage disequilibrium < 30%. Bootstrap values are provided in Supplemental Figure S5. (**B**) Population structure and admixture as determined by STRUCTURE^68^ analysis of the 90 ATGC *Chenopodium* accessions using the Puechmaille method^70^ with *K* = 8 groups. Accession groups are labeled as follows: BERL = *C. berlandieri* var. *berlandieri*; BOSC = *C. berlandieri* var. *boscianum*; MACR = *C. berlandieri* var. *macrocalycium*; NUTT = *C. berlandieri* subsp. *nuttaliae*; SINU = *C. berlandieri* var. *sinuatum*; ZSCH 1 = *C. berlandieri* var. *zschackei* Group 1; ZSCH 2 = *C. berlandieri* var. *zschackei* Group 2; QUIN = *C. quinoa* subsp. *quinoa*, *C. quinoa* subsp. *melanosperma*, and *C. hircinum*.

A panel of 90 AABB tetraploid *Chenopodium* genotypes, representing the diversity within the ATGC (Supplemental Table S1 and Supplemental Fig. S6), was resequenced using Illumina short reads to produce a total 1.29 Tb of sequence, with an average coverage of 5.25X per genotype after trimming and quality control. An initial SNP dataset consisting of 8,195,026 SNPs with no missing data was further pruned by SNPhylo based on minor allele frequency and linkage disequilibrium to produce a final data set consisting of 12,915 SNPs, with an average of 718 SNP per chromosome. IQ-TREE was used to build a maximum likelihood estimation tree based on a GTR + G + AS model which yielded a robust tree (Fig. 3A; Supplemental Fig. S7). All major branch nodes of the tree exhibited high (> 95% confidence) bootstrap values, with the exception of one node within the North American *C. berlandieri* var. *zschackei* branch toward the base of the tree. All *C. berlandieri* var. *nuttaliae* accessions occupied a single branch of the tree, at the base of which was a black-seeded accession, BYU 1486, which is probably a sample of what was previously identified as wild-weedy *C. pueblense*^15^ and according to this analysis clearly does not merit classification as a separate species. Basal to this branch of the tree is quinoa variety 0654, which appears to be derived from hybridization between quinoa and huauzontle. A second major branch includes samples of wild-weedy and cultivated South American ATGC members, among them Andean quinoas and free-living *ajara* (black-seeded weedy or feral types, classified as *C. quinoa* subsp. *melanosperma*^7^) with a separate sub-branch containing the coastal quinoa varieties. Black-seeded hircinum or melanosperma types from irrigated valleys of the Pacific Slope of the Andes – BYU 1904 from Arequipa and BYU 566 from Tarapacá – grouped basally with Highland and Chilean coastal quinoas, respectively, indicating possible ancestral relationships with each of these two cultivated groups. Situated proximally, at the base of this long branch characteristic of a domestication bottleneck, were wild-weedy samples of *C. hircinum* from the Chilean Central Valley (BYU 17127) and the Argentine Pampas (BYU 1101 and 1770).

All of the tree branches basal to these domesticated groups consist of accessions of free-living *C. berlandieri* from North America. The closest branch to the domesticated groups had two sub-branches: one consisting of eastern Oklahoma var. *sinuatum*, New England coastal var. *macrocalycium*, and a lone sample of the ENA floodplain ecotype from the Mississippi River drainage (BYU 1312) of var. *bushianum*; and a second sub-branch consisting of Gulf of Mexico coastal var. *boscianum* and South Texas var. *berlandieri* ecotypes. Moving closer to the base of the tree, the next branch consisted of accessions from the southwestern low-elevation deserts and Mediterranean coastal zone of California (Group ZSCH 2) that would morphologically be categorized as a mixture of vars. *sinuatum* and *zschackei*. At the base of the tree was a large and highly diverse branch consisting of Great Basin, Rocky Mountain, and western Great Plains strains of var. *zschackei* (Group ZSCH 1).

A STRUCTURE analysis was performed on the 90 genotypes using the Puechmaille method^70^ which is d**e**signed to find hidden structure and gene flow while reducing bias associated with uneven population sampling. STRUCTURE predicted *K* = 8 groups, which mirrored the results of the phylogenetic analyses. In Fig. 3B, the eight groups are labeled as follows: zschackei Group 1 (brown, ZSCH 1); nuttaliae (blue, NUTT); quinoa and hircinum (gold, QUIN); boscianum (orange, BOSC); macrocalycium (yellow, MACR); Plains sinuatum (purple, SINU); Southwest sinuatum-zschackei Group 2 (pink, ZSCH 2); and South Texas berlandieri (salmon, BERL). The high level of inter-group admixture within the ZSCH 1 group and, to a lesser extent, ZSCH 2, suggests this botanical variety represents a taxonomic catch-all for highly diverse *C. berlandieri* strains found throughout western North America. In contrast, the SINU, MACR, BOSC, and BERL groups of free-living *C. berlandieri* are relatively cohesive, showing little to no admixture from other groups. Interestingly, the Mississippi River floodplain strain of var. *bushianum*, BYU 1312, exhibited genetic components from all eight Puechmaille groups. It remains to be seen if an expanded genotypic analysis including a larger number of *C. berlandieri* var. *bushianum* accessions from ENA would reveal the existence of a ninth ATGC ecotype.

The two domesticated ATGC groups are enriched for unique alleles that were undoubtedly the result of selection within the intermediate-elevation agricultural environment of South-Central Mexico and Andean South America for the NUTT and QUIN groups, respectively. Significantly, all three low-elevation hircinum types within the QUIN group displayed similar North American genetic signature components, the largest from the South Texas BERL group, potentially hinting at a southern Great Plains origin of the South American ATGC. Similarly, the black-seeded Mexican strain BYU 1486 contained slight genetic signatures of the BERL and MACR groups. The other anomaly in the dataset is quinoa variety 0654, which contained roughly equivalent genomic contributions from quinoa and huauzontle.

## Discussion

The whole-genome assembly of *C. berlandieri* subsp. *nuttaliae* PI 433231 (accession BYU 1484) reveals a close genetic relationship between this domesticated Mesoamerican chenopod and Andean quinoa, which most likely share a common A-genome cytoplasm^78^ and AABB ancestor^9,12,19^. However, phylogenetic (Fig.3A) and STRUCTURE (Fig. 3B) analyses indicate that these two taxonomic entities most likely arose through separate domestication events and remained separated, as was likely the case with the amaranths, another set of secondary crop-pseudocereals of New World origin^85–87^. These results contrast with maize and tomato, primary crops whose evolution as domesticates involved geographic translocation between their Mesoamerican and Andean centers of origin, respectively^88–90^.

Huauzontle accession PI 433231 from Mesoamerica and QQ74 quinoa from the Chilean Central Valley differ by two large inversions (3B, 4B), a complex series of rearrangements on 5A, and a 4A-6B reciprocal translocation (Fig. 1). Moreover, the pericentric 3B inversion in the Chilean QQ74 reference genome, while spanning well over half the physical length of the chromosome, represents a relatively modest linkage distance of ~40cM, roughly one-third of the linkage map (Supplemental Fig. S4). This inversion was previously detected in a subset of quinoas of lowland Chilean origin^10^ and did not appear to be fixed in any particular lineage. These results suggest that this inversion likely has only minor effects on hybrid fertility and is not a species-delineating structural variant. Moreover, the abundant recombination and relative fertility in the Real-1 × BYU 937 F_2_ population (Fig. 1A) suggest that at least some ecotypes of *C. berlandieri*—for example, Gulf Coast var. *boscianum*—may provide unique breeding resources for improving quinoa, especially its tolerance to heat, flooding, and lowland pests and diseases^3^. At the same time, the extensive degree of chromosomal rearrangement differentiating PI 433231 from the quinoas would complicate a breeding scheme to transfer the former’s mutant *GBSSI* alleles for high-amylopectin seed starch—a potentially valuable culinary characteristic—into Andean quinoas via sexual crossing^22,35^.

Other potentially interesting structural variants are the telomeric inversions on chromosomes 4A in Real-1 and 6A in *C. hircinum* BYU 1101 (Fig. 1A). Previously, putative telomeric inversions were detected in whole-genome assemblies of the A-genome diploid *C. watsonii*, in comparison with the South American diploid *C. pallidicaule* and QQ74^60^. The AA diploids adaptively radiated into >30 taxonomic species throughout the Americas and in many cases form populations that are not only sympatric but also synchronously flowering. Interspecies hybrid fertility should conceivably be decreased through crossovers within distal inverted segments in heterozygotes. Alternatively, telomeric inversions in such hybrids could potentially restrict recombination to the non-inverted arm, thus enhancing haploblocks along the entire unpaired arm. Consequently, we intend to further investigate whether a propensity for chromosomal differentiation through telomeric inversions might be a unique evolutionary characteristic of the *Chenopodium* A genome—and may have driven an adaptive radiation within this group. Also, further deep sequencing of BYU 937 and other ecotypes of *C. berlandieri* will be helpful in resolving the exact nature of the structural differences driving the evolution of the nine pitseed goosefoot subspecies/botanical varieties and in identifying ideal crossing partners for quinoa improvement.

Analyses of the resequencing panel of ATGC accessions via SNP markers provided insights into the complex ecotypic structure of what is arguably a single allotetraploid biological species, *C. quinoa* Willd., with a native range from the Arctic Ocean to Tierra del Fuego and consisting of wild, weedy, and multiple domesticated sub-taxa. The Puechmaille model predicted eight, and possibly a ninth with BYU 1312, unique ATGC entities (Fig. 3B), along with multiple admixed intermediates within a “catch-all” taxon, *C. berlandieri* var. *zschackei*. While the Andean (QUIN) and Mesoamerican (NUTT) cultigens form distinct groups, low-elevation *C. hircinum* strains within the former group have significant genetic components in common with southern Great Plains *C. berlandieri*, and especially with the South Texas strains of the BERL group (BYU 1804, 1821, 1831), suggesting that region of North America might have been the source of the South American ATGC via long-range dispersal. Young et al.^60^ provided evidence for intra-hemispheric dispersal of *Chenopodium* diploids between the southern Great Plains/South Texas and northern Argentina, hypothesizing that this may have occurred multiple times in antiquity via seasonal avian migration. Interestingly, all the accessions in the BERL group have the foul-smelling TMA phenotype—in common with *C. hircinum* accessions from the QUIN group (BYU 1101, 1770, 17127). This same pattern of allele sourcing reveals *C. hircinum* as the likely source of the QUIN group, as expected and previously suggested by numerous sources^1,4,6,7,9^, with the Pacific Slope strains BYU 566 (Tarapacá, Chile, 1533 meters elevation) and BYU 1904 (Arequipa, Perú, 2,208 meters elevation) showing evidence for ancestral relationships with coastal Chilean (the former) and Andean (the latter) domesticated quinoas. Also noteworthy is that two accessions of free-living *C. quinoa* subsp. *melanosperma* (BYU 1777 and 1789, both from the Lake Titicaca Basin) were embedded within the domesticated quinoas and not at their root, suggesting these represent endoferalized strains of quinoa and not semi-wild ancestors^13^.

The phylogenetic and STRUCTURE analyses (Fig. 3) indicate the area with the greatest diversity for the ATGC is western North America. Within this region are mostly semi-arid, arid, and Mediterranean climates, at altitudes ranging from sea level to approximately 3,500 meters; consequently, populations of *C. berlandieri* here, representing groups ZSCH 1 and ZSCH 2, should possess extensive variation for abiotic stress tolerance. We would propose that taxonomists consider a new botanical variety name for the genetically distinct ZSCH 2 accessions from the North American Southwest, perhaps var. *wilsonii* in honor of Hugh Daniel Wilson, the late botanist at Texas A&M University who devoted much of his career to studying genetic relationships within the ATGC and was an American Association for the Advancement of Science Fellow. At least one southeastern North American population of *C. berlandieri* var. *boscianum*, BYU 937 from Galveston Bay, Texas, has retained a high enough level of chromosomal homology with Bolivian quinoa Real-1 to produce hybrid progeny with highly faithful chromosome pairing and, consequently, high fertility and fecundity. Efforts to hybridize these and other strains of *C. berlandieri* with *C. quinoa*, to produce breeding populations segregating for environmental stress resistance genes from the former and agronomic quality genes from the latter, are well underway. These populations are being freely shared with quinoa breeding programs targeting lowland tropical, subtropical, and warm-season temperate production environments on five continents in the hope that quinoa may soon be incorporated as a staple into diets to alleviate malnutrition and improve worldwide food security in the face of global climate change. This study reveals the tremendous potential of *C. berlandieri* as a quinoa breeding partner, both for improving lowland warm-season (including subtropical and tropical) abiotic traits and for enhancing overall diversity within the quinoa breeding pool.

### Supplementary Information

Supplementary Information.

## Data Availability

Raw PacBio (genome, SRR26349240-SRR26349241) and Illumina (RNAseq, SRR26349235-SRR26349238; polishing, SRR26349239; Hi-C, SRR26363924, GBS, SRR26366783-SRR26367027, resequencing, SRR26340453- SRR26340542) reads have been deposited in GenBank under BioProject ID PRJNA1026646 (https://dataview.ncbi.nlm.nih.gov/object/PRJNA1026646). A complete listing of raw data deposited in NCBI is provided in Supplemental Table S3. The complete genome has been deposited at DDBJ/ENA/GenBank under the accession JAWIZW000000000. The genome and annotation is also available on the Comparative Genomics (CoGe) platform of CyVerse as Genome ID# 62,441 (https://genomevolution.org/coge/). The CoGe platform allows users to download bulk data (genome + annotation), view the genome using JBrowse and perform a variety of comparative genome analyses, including blast analyses and multispecies synteny comparisons.
