# Supplementary material for: North American pitseed goosefoot (Chenopodium berlandieri) is a genetic resource to improve Andean quinoa (C. quinoa)
Source: Sci Rep. 2024 May 29;14:12345. doi: 10.1038/s41598-024-63106-8 (PMC11137100; doi:10.1038/s41598-024-63106-8)
Supplement: Supplementary file 1 — Supplementary Information. [file 41598_2024_63106_MOESM1_ESM.docx]

**­­­­Supplemental Figure S1:** Bash and Python scripts for High Confidence / Low Confidence gene model classification.

1. Blast annotated protein sequences against the TrEMBL database. To speed things up, you can split the protein.fasta file into smaller chunks and run a for loop:

#!/bin/sh

scripts_dir=./scripts

database_dir=./databasesHCLC/db

database=TrEMBL_taxID_3398_Magnoliopsida_13244806proteins.fasta

for file in *fasta

do

name=`echo $file | sed 's/.fasta//'`

mkdir ${scripts_dir}/${name}

cat > ${scripts_dir}/${name}/${name}.sh <<EOF

#!/bin/bash

#SBATCH --time=72:00:00 # walltime

#SBATCH --ntasks=24 # number of processor cores (i.e. tasks)

#SBATCH --nodes=1 # number of nodes

#SBATCH --mem-per-cpu=4G # memory per CPU core

#SBATCH -J "$name" # job name

module purge

module load conda-pws

module load blast_2.12

makeblastdb -in ${database_dir}/${database} -input_type fasta -dbtype prot && ##the first time you do this you need to make a blastdb with this portion of the script.

blastp -db ${database_dir}/${database} -query ${file} -out ${file}_v_${database}.blasp -evalue 0.000001 -outfmt "6 qseqid sseqid evalue slen qlen length pident qcovs" -num_alignments 1 -seg yes -soft_masking true -lcase_masking -max_hsps 1 -num_threads 24

EOF

sbatch ${scripts_dir}/${name}/${name}.sh

done

**Once this finishes you’ll need to concatenate the blasp results together to make a single, final, file.

cat *.blasp > peptide_v_TrEMBL_taxID_3398_Magnoliopsida_proteins.fasta.blasp

1. Blast the sequences against the repeat database from TREP. Since this database is small, Blast the whole protein file at once.

#!/bin/bash

#SBATCH --time=72:00:00 # walltime

#SBATCH --ntasks=24 # number of processor cores (i.e. tasks)

#SBATCH --nodes=1 # number of nodes

#SBATCH --mem-per-cpu=4G # memory per CPU core

#SBATCH -J "blastp" # job name

#SBATCH --mail-type=FAIL

module purge

module load conda-pws

module load blast_2.12

data_dir=./databasesHCLC/db

query=$1 ##peptide sequences from annotation

database=trep-db_proteins_Rel-19.fasta ##this is stored in the db directory

makeblastdb -in ${data_dir}/${database} -input_type fasta -dbtype prot && blastp -db ${data_dir}/${database} -query ${query} -out ${query}_v_${database}.blasp -evalue 0.000001 -outfmt "6 qseqid sseqid evalue slen qlen length pident qcovs" -num_alignments 1 -seg yes -soft_masking true -lcase_masking -max_hsps 1 -num_threads 24

1. Segregate the protein into High Confidence and Low Confidence annotations using the auto_calc_filter_gff.sh script. This script calls two Python scripts that must be in your working directory: 1) calc_blastp.py and 2) filter_script3.py. These scripts can be found below.

There are parameters you can change in the **filter_script3.py** on ln 11 and 12 – make sure to copy those same values to the auto_calc_filter_gff.sh below. They need to be the same in both scripts.

auto_calc_filter_gff.sh

#!/bin/sh

##useage: sh auto_calc_filter_gff.sh

##It is assumed that you already have the blast outputs from above for the TrEMBL and TREP blast output.

##Place the following scripts in the working directory: calc_blastp.py and filter_script3.py and the busco5_protein.sh

blastp=peptide_v_TrEMBL_taxID_3398_Magnoliopsida_proteins.fasta.blasp ##The TrEMBL blast results

original_pep_file=peptide.pep ##from annotation (Maker or another annotator)

original_gff_file=Maker.gff ##original gff file from the de novo annotation

**percentID=66 ##a good starting value is 66 – Make sure to have the same value in line 12 of the filter_script3.py executable!!**

**ratio_cutoff=2.2 ##a good starting value is 2.2 – Make sure to have the same value in line 11 of the filter_script3.py executable 11!!**

TREP_blastp=peptide_v_trep-db_proteins_Rel-19.fasta.blasp ##The TREP blast results

##add the additional columns (qlen/slen, etc) to the SWISSPROT.blastp output

python3 calc_blastp.py ${blastp} ${blastp}_output &&

echo -e "\nRunning filter against the Swiss-Prot database"

##filter to identify the HC gene models

python3 filter_script3.py ${blastp}_output ${blastp}_output_fail_${ratio_cutoff}_${percentID}.list ${blastp}_output_pass_${ratio_cutoff}_${percentID}.list &&

##make a new protein file of the HC peptides that meet the criteria for the filter (Pid, qcov and scov, etc.)

seqtk subseq ${original_pep_file} ${blastp}_output_pass_${ratio_cutoff}_${percentID}.list > passEvalue_qcovscov_Pid_${blastp}_${ratio_cutoff}_${percentID}_HC.pep &&

##add the additional columns (qlen/slen, etc) to the TREP.blastp output

python3 calc_blastp.py ${TREP_blastp} trep.out &&

echo -e "\nRunning filter against the TREP database"

##filter to identify high quality hits to the TREP database (we use the same parameter for both the TREP and the SWISSprot)

python3 filter_script3.py trep.out TREP_failed.list TREP_passed.list

##Remove from the above protein file any that match the TREP database (these were previously identified with blastp and those with hits to the TREP db or in the TREP_passed.list

module load seqkit

seqkit grep -v -f TREP_passed.list passEvalue_qcovscov_Pid_${blastp}_${ratio_cutoff}_${percentID}_HC.pep -j 12 -o passTREP_Evalue_qcovscov_Pid_${blastp}_${ratio_cutoff}_${percentID}_HC.pep2 &&

##Report to the screen the number of HC gene models left after the remove of the TREP protein matches

echo ""

echo -n "Number HC gene models remaining after TREP removal: "

count_HC=$(grep ">" -c passTREP_Evalue_qcovscov_Pid_${blastp}_${ratio_cutoff}_${percentID}_HC.pep2)

echo "$count_HC"

##make as list of the protein names in the HC protein file (_HC.pep2). You need two list, one with the -RA one without

grep ">" passTREP_Evalue_qcovscov_Pid_${blastp}_${ratio_cutoff}_${percentID}_HC.pep2 | cut -d " " -f1 | sed 's/>//g ; s/-RA//g' > HC_${ratio_cutoff}_${percentID}.list &&

grep ">" passTREP_Evalue_qcovscov_Pid_${blastp}_${ratio_cutoff}_${percentID}_HC.pep2 | cut -d " " -f1 | sed 's/>//g' > HC_${ratio_cutoff}_${percentID}-RA.list &&

##Make the HC gff file

grep -Ff HC_${ratio_cutoff}_${percentID}.list ${original_gff_file} | sort -k1,1 -k4,4n > ${original_gff_file}_HC_${ratio_cutoff}_${percentID}.gff &&

##report some stats for the HC.gff

echo -e "\nstats for HC.gff:"

cut -f3 ${original_gff_file}_HC_${ratio_cutoff}_${percentID}.gff | sort | uniq -c

##Make a protein file for the LC protein. We are doing this by inverse grep with the original protein file and the HC-RA.list

seqkit grep -v -f HC_${ratio_cutoff}_${percentID}-RA.list ${original_pep_file} > failTREP_Evalue_qcovscov_Pid_${blastp}_${ratio_cutoff}_${percentID}_LC.pep2

##Report to the screen the number of LC gene models

echo ""

echo -n "Number LC gene models remaining: "

count_LC=$(grep ">" -c failTREP_Evalue_qcovscov_Pid_${blastp}_${ratio_cutoff}_${percentID}_LC.pep2)

echo "$count_LC"

##make as list of the protein names in the LC protein file (_LC.pep2). You need two list, one with the -RA one without

grep ">" failTREP_Evalue_qcovscov_Pid_${blastp}_${ratio_cutoff}_${percentID}_LC.pep2 | cut -d " " -f1 | sed 's/>//g ; s/-RA//g' > LC_${ratio_cutoff}_${percentID}.list &&

grep ">" failTREP_Evalue_qcovscov_Pid_${blastp}_${ratio_cutoff}_${percentID}_LC.pep2 | cut -d " " -f1 | sed 's/>//g' > LC_${ratio_cutoff}_${percentID}-RA.list &&

##Make the LC.gff

grep -Ff LC_${ratio_cutoff}_${percentID}.list ${original_gff_file} | sort -k1,1 -k4,4n > ${original_gff_file}_LC_${ratio_cutoff}_${percentID}.gff &&

##Report some stats for the LC.gff

echo -e "\nstats for LC.gff:"

cut -f3 ${original_gff_file}_LC_${ratio_cutoff}_${percentID}.gff | sort | uniq -c

##cleanup

mkdir results_${ratio_cutoff}_${percentID}

mv ${blastp}_output results_${ratio_cutoff}_${percentID}/

mv ${blastp}_output_fail_${ratio_cutoff}_${percentID}.list results_${ratio_cutoff}_${percentID}/

mv ${blastp}_output_pass_${ratio_cutoff}_${percentID}.list results_${ratio_cutoff}_${percentID}/

mv passEvalue_qcovscov_Pid_${blastp}_${ratio_cutoff}_${percentID}_HC.pep results_${ratio_cutoff}_${percentID}/

mv passTREP_Evalue_qcovscov_Pid_${blastp}_${ratio_cutoff}_${percentID}_HC.pep2 results_${ratio_cutoff}_${percentID}/

mv HC_${ratio_cutoff}_${percentID}.list results_${ratio_cutoff}_${percentID}/

mv HC_${ratio_cutoff}_${percentID}-RA.list results_${ratio_cutoff}_${percentID}/

mv ${original_gff_file}_HC_${ratio_cutoff}_${percentID}.gff results_${ratio_cutoff}_${percentID}/

mv failTREP_Evalue_qcovscov_Pid_${blastp}_${ratio_cutoff}_${percentID}_LC.pep2 results_${ratio_cutoff}_${percentID}/

mv LC_${ratio_cutoff}_${percentID}.list results_${ratio_cutoff}_${percentID}/

mv LC_${ratio_cutoff}_${percentID}-RA.list results_${ratio_cutoff}_${percentID}/

mv ${original_gff_file}_LC_${ratio_cutoff}_${percentID}.gff results_${ratio_cutoff}_${percentID}/

mv trep.out results_${ratio_cutoff}_${percentID}/

mv TREP_failed.list results_${ratio_cutoff}_${percentID}/

mv TREP_passed.list results_${ratio_cutoff}_${percentID}/

Python scripts:

1) calc_blastp.py:

import sys

import pandas as pd

def main(input_file_path, output_file_path):

# Load the text file into a DataFrame

df = pd.read_csv(input_file_path, delimiter='\t', header=None) # Adjust delimiter if needed

# Set column headers

df.columns = ["qseqid", "sseqid", "evalue", "slen", "qlen", "length", "pident", "qcovs"]

# Calculate additional columns

df["qcov"] = (df["length"] / df["qlen"]).round(4)

df["scov"] = (df["length"] / df["slen"]).round(4)

df["qlen/slen"] = (df["qlen"] / df["slen"]).round(4)

df["qcov/scov"] = (df["qcov"] / df["scov"]).round(4)

df["qcov/scov + qlen/slen"] = (df["qlen/slen"] + df["qcov/scov"]).round(4)

# Save the modified DataFrame back to a tab-delimited text file with headers

df.to_csv(output_file_path, sep='\t', header=True, index=False)

if __name__ == "__main__":

if len(sys.argv) != 3:

print("Usage: python script.py input_file_path output_file_path")

else:

input_file = sys.argv[1]

output_file = sys.argv[2]

main(input_file, output_file)

2) filter_script3.py (values that should be sync’d to the auto_calc_filter_gff.sh are on lines 11 & 12).

import sys

import pandas as pd

def main(input_file_path, output_failed_values_path, output_passing_values_path):

# Load the text file into a DataFrame

df = pd.read_csv(input_file_path, delimiter='\t', header=0) # Adjust delimiter if needed

# Filter rows based on conditions ###use 1.6 and 2.5 for 25% divergence; 1.67 and 2.4 for 20%; 1.74 and 2.3 for 15%; 1.81 and 2.2 for 10% for the second parameter

failed_df = df[

(df["evalue"] > 1.0E-10) |

((df["qcov/scov + qlen/slen"] < **1.81**) | (df["qcov/scov + qlen/slen"] > **2.2**)) |

(df["pident"] <= **66**)

]

# Calculate the number of rows that passed and failed the filter

num_passed_rows = len(df) - len(failed_df)

num_failed_rows = len(failed_df)

# Print the number of rows that passed and failed the filter

print(f"Number of gene models that passed the filter: {num_passed_rows}")

print(f"Number of gene models that failed the filter: {num_failed_rows}")

# Write the list of failed values to the output file

failed_values = failed_df["qseqid"].tolist()

with open(output_failed_values_path, 'w') as output_failed_values:

for value in failed_values:

output_failed_values.write(value + '\n')

# Write the list of passing values to the output file

passing_df = df.drop(failed_df.index)

passing_values = passing_df["qseqid"].tolist()

with open(output_passing_values_path, 'w') as output_passing_values:

for value in passing_values:

output_passing_values.write(value + '\n')

if __name__ == "__main__":

if len(sys.argv) != 4:

print("Usage: python script.py input_file_path output_failed_values_path output_passing_values_path")

else:

input_file = sys.argv[1]

output_failed_values = sys.argv[2]

output_passing_values = sys.argv[3]

main(input_file, output_failed_values, output_passing_values)

1. Lastly make the high LC and HC gff, pep and transcript files (example below is for the LC gene models):

Concatenate all the gene IDs together with the nested features:

cat LC_2.2_66.list LC_2.2_66-RA.list > LC_2.2_66all.list

then, use grep to pull out all of those genes/nested features:

grep -Ff LC_2.2_66all.list Final_allscaffolds_functional_blast.genes_includestRNAs.AGAT.gff | sort -k1,1 -k4,4n > LC.gff

Then sort this and fix with AGAT (https://github.com/NBISweden/AGAT):

agat_convert_sp_gxf2gxf.pl --gff LC.gff --output LC.genes.AGAT.gff

Pull the proteins (pep) and transcripts (cds) files:

agat_sp_extract_sequences.pl -g LC.genes.AGAT.gff -f FINAL_AeU_asm.fasta -p --output LC.AGAT.pep

agat_sp_extract_sequences.pl -g LC.genes.AGAT.gff -f FINAL_AeU_asm.fasta -t cds --output LC.AGAT.cds

Make specific gff files for the HC and LC gene models:

agat_sp_filter_feature_from_keep_list.pl --gff brakerRNAref_IsoSeqcomp_functional.gff3 --keep_list LC_1.5_2.67_66.list --output LC_brakerRNAref_IsoSeqcomp_functional.gff3

Supplemental Figure S2: BUSCO results for *C. berlandieri* subsp. *nuttaliae* genome, annotated transcriptome and annotated protein using the Embryophyta odb10 database. BUSCO results are also provided for high- and low-confidence categorization of the gene models. High COG duplication is expected for this tetraploid species.

**Supplemental Figure S3:** Cumulative frequency of gene models based on the Annotation Edit Distance (AED) calculated by MAKER^53^. AED measures the quality of a gene annotations relative to specificity and sensitivity. MAKER derived gene annotations (red) were categorized as high confident (blue) or low confident (gray).

**Supplemental Figure S4:** A 3B pericentric inversion identified in *C. quinoa* var. QQ74^10^. (A) Collinearity analysis of the physical map of chromosome 3B in *C. quinoa* and the linkage map for the interspecific Real-1 × *C. berlandieri* var. *boscianum* accession ‘BYU 937’ F_2_ population. (B) A Circos plot of tandem repeats identified for the *C. quinoa* genome assembly. The blue arrow in both figures identifies the approximate position of the centromere based on the density of the monomer of the pericentric repeat in chromosome 3B (40 – 43 Mb) as identified by TRASH^77^.

**Supplemental Figure S5.** (A) The assembled and annotated *C*. *berlandieri* subsp. *nuttaliae* chloroplast genome. (B) Representation of the inheritance of the extranuclear genomes in Chenopodium. Tick marks represent 2 Kb. The chloroplast (Cp; MK159176.1) and mitochondria (Mt; MK182703.1) genomes are represented on the outside track, while the genotype call (red: adenine, yellow: cytosine, blue: guanine, and green: thymine) for the maternal, F_1_ hybrid and paternal parent are provided in tracks 2 – 4, respectively. SNP calling was excluded in the inverted repeat region of the chloroplast. Red arrows identify two SNPs (out of 404) that were identified as paternally inherited.

**Supplemental Figure S6**. Geographic distribution of ATGC accessions collected in the USA and included in the diversity panel. Additional passport information is provided in Supplemental Table S1. Stars represent collected populations, as follows: red = *C. berlandieri* var. *berlandieri*; blue = *C. berlandieri* var. *boscianum*; gray = *C. berlandieri* var. *macrocalycium*; purple = *C. berlandieri* var. *sinuatum* (Southern Plains ecotype); yellow = *C. berlandieri* var. *zschackei* Clade 1; turquoise = *C. berlandieri* var. *zschackei* Clade 2; white = undefined *C. berlandieri* variety from river bottoms of the Eastern Woodlands. The map was generated using the online version of Google Maps, https://www.google.com/maps/d/u/0/edit?hl=en&mid=13sT012zvitjal1_fOs2Rc6KV_XtTVQ0&ll=33.29800856497302%2C-95.497834566434&z=5.


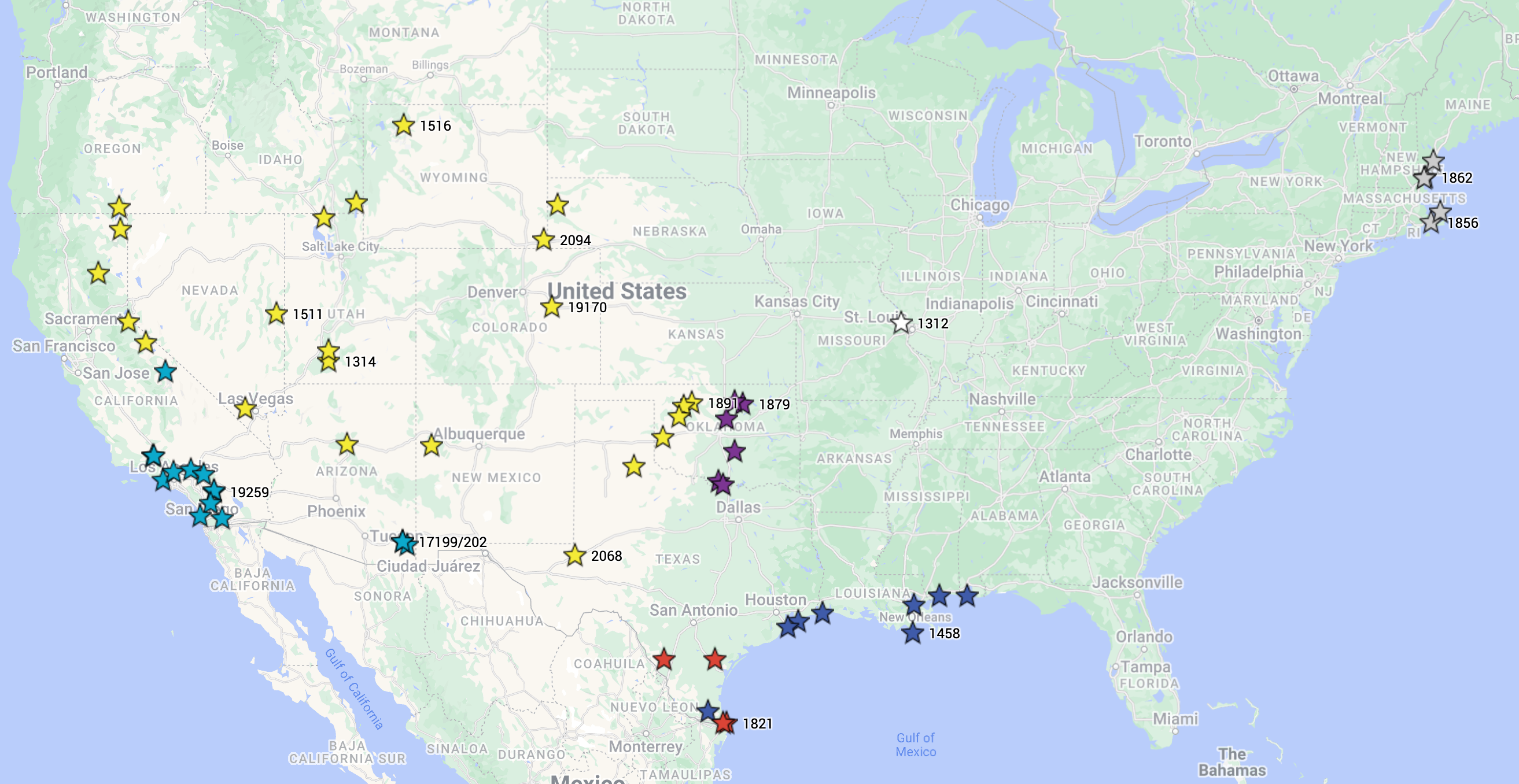


**Supplemental Figure S7**. An MLE-based tree of 90 ATGC accessions from Supplemental Table S1. Bootstrap values from IQ-Tree nodes based on SH-aLRT support values. Passport information is in Supplemental Table S2. Each color denotes a different clade delineation, as per the STRUCTURE analysis (Fig. 3).





ZSCH 1

ZSCH 2

BERL

BOSC

MACR

SINU

QUIN

NUTT

**Supplemental Table S1**. ATGC accessions included in this study which included 16 genotypes of *C. quinoa*; five of *C. hircinum*; 17 strains of *C. berlandieri* ssp. *nuttaliae*; and 52 accessions of *C. berlandieri* ssp. *berlandieri*. Genotypic designations based on the STRUCTURE and MLE clades (Figure 3) are also indicated.

| ***Chenopodium* Taxon** | **BYU Number** | **Genotype/ Clade** | **Other Accession ID** | **Origin** |
| --- | --- | --- | --- | --- |
| *quinoa* subsp. *quinoa* | 1439 | QUIN | Cherry Vanilla | Wild Garden Seed, Philomath, Oregon, USA |
|  | 1650 | QUIN | Chucapaca; PI 674265 | Patacamaya, Bolivia |
|  | 1613 | QUIN | CICA-17 | FAO-CIP International Quinoa Nursery (A. Mujica) |
|  | 1624 | QUIN | G-205-95-DK | FAO-CIP International Quinoa Nursery (A. Mujica) |
|  | 2044 | QUIN | KU-2 | FAO-CIP International Quinoa Nursery (A. Mujica) |
|  |  | QUIN | LP (Kurmi) | Bolivia (A. Bonifacio) |
|  | 1615 | QUIN | Ollague | FAO-CIP International Quinoa Nursery (A. Mujica) |
|  | 1202 | QUIN | Pasankalla | Bolivia-Perú |
|  | 1633 | QUIN | Real-1 | FAO-CIP International Quinoa Nursery (A. Mujica) |
|  | 947 | QUIN | Regalona | Semillas Baer, Temuco, Chile |
|  | 1842 | QUIN | Salcedo-INIA | Puno, PFAO-CIP International Quinoa Nursery (A. Mujica) |
|  |  | QUIN | UDEC-2; PI 634921 | Valdivia, Los Ríos, Chile |
|  | 1648 | NA | 0654 | Puno, Perú (A. Mujica) |
|  | 1662 | QUIN | D. Oros | ININ, Ocoyoacac, México, México |
| *quinoa* subsp. *melanosperma* | 1777 | QUIN |  | Atuncolla, Puno, Perú |
|  | 1789 | QUIN |  | Acochupa, Puno, Perú |
| *hircinum* | 566 | QUIN |  | Tarapacá Valley, Tarapacá, Chile |
|  | 1101 | QUIN |  | Ceres, Santa Fé, Argentina |
|  | 1770 | QUIN |  | Senillosa, Neuquén, Argentina |
|  | 17127 | QUIN |  | Lolol, O’Higgins, Chile |
|  | 1904 | QUIN |  | Jacobo Hunter District, Arequipa, Perú |
| *berlandieri* subsp. *nuttaliae* | 567 | NUTT |  | Opopeo, Michoacán, México |
|  | 668 | NUTT |  | Tecomán, Colima, México |
|  | 1447 | NUTT | H-03 | ININ, Ocoyoacac, México, México |
|  | 1483 | NUTT | PI 433230 | Guadalajara, Jalisco, México |
|  | 1484 | NUTT | PI 433231 | Atlixco, Puebla, México |
|  | 1485 | NUTT | PI 568155 | Cacaloxuchil, Puebla, México |
|  | 1486 | NUTT | PI 568156 | Santiago Acutzilapan, México, México |
|  | 1647 | NUTT | H-04 |  |
|  | 1663 | NUTT | Zumbaro | Santa Maria Huramangaro, Michoacán, México |
|  | 1664 | NUTT | J. Silva | Michoacán, México |
|  | 1666 | NUTT | P. Bravo | Michoacán, México |
|  | 1668 | NUTT | H-16 | La Concepcion Huichochitlan, México, México |
|  | 1669 | NUTT | H-18 | ININ, Ocoyoacac, México, México |
|  | 17176 | NUTT | Red Aztec Spinach | Wild Garden Seed, Philomath, Oregon, USA |
|  | 19284 | NUTT | Kelite | México (A. Bonifacio) |
|  | 19286 | NUTT | H-06 #2 | ININ, Ocoyoacac, México, México |
|  | 19287 | NUTT | H-07 #2 | El Capulin, Otzolotepec, México, México |
| *berlandieri* subsp. *berlandieri* | 1312 | NA |  | St. Charles, Missouri, USA |
|  | 1314 | ZSCH 1 |  | Hatch, Utah, USA |
|  | 14108 | ZSCH 2 | Ames 33013 | Witch Creek, Arizona, USA |
|  | 1891 | ZSCH 1 |  | Aline, Oklahoma, USA |
|  | 1896 | ZSCH 1 |  | Waynoka, Oklahoma, USA |
|  | 1897 | ZSCH 1 |  | Lenora, Oklahoma, USA |
|  | 1965 | ZSCH 1 |  | Cedarville, California, USA |
|  | 19127 | ZSCH 2 | Ames 35352 | Bishop, California, USA |
|  | 19146 | ZSCH 1 | Ames 35354 | Bridgeport, California, USA |
|  | 19239 | ZSCH 2 |  | San Bernardino Mountains, California, USA |
|  | 19259 | ZSCH 2 | Ames 35355 | San Jacinto Mountain, California, USA |
|  | 19302 | ZSCH 2 |  | Lake of the Woods, California, USA |
|  | 19306 | ZSCH 2 |  | Palomar Mountain, California, USA |
|  | 2004 | ZSCH 2 |  | Laguna Mountains, California, USA |
| *berlandieri* subsp. *berlandieri* var. *berlandieri* | 1804 | BERL |  | Catarina, Texas, USA |
|  | 1819 | BOSC |  | Lasara, Texas, USA |
|  | 1831 | BERL |  | Skidmore, Texas, USA |
| *berlandieri* subsp. *berlandieri* var. *boscianum* | 937 | BOSC |  | Texas City, Texas, USA |
|  | 1458 | BOSC | PI 698414 | Golden Meadow, Louisiana, USA |
|  | 1465 | BOSC | PI 698420 | Little Florida Beach, Louisiana, USA |
|  | 1469 | BOSC | PI 698423 | Bolivar Peninsula, Texas, USA |
|  | 14111 | BOSC | PI 698427 | Mobile Bay, Alabama, USA |
|  | 14112 | BOSC | PI 698428 | Long Beach, Mississippi, USA |
|  | 17132 | BOSC |  | New Orleans, Louisiana, USA |
|  | 1821 | BERL |  | Port Isabel, Texas, USA |
| *berlandieri* subsp. *berlandieri* var. *macrocalycium* | 803 | MACR | PI 666279 | Saco, Maine, USA |
|  | 1856 | MACR |  | Sandwich, Massachusetts, USA |
|  | 1858 | MACR |  | Truro, Massachusetts, USA |
|  | 1862 | MACR |  | Periwinkle Cove, New Hampshire, USA |
|  | 1863 | MACR |  | Rye Harbor, New Hampshire, USA |
| *berlandieri* subsp. *berlandieri* var. *sinuatum* | 1511 | ZSCH 1 |  | Sacramento Pass, Nevada, USA |
|  | 17199 | ZSCH 2 |  | Wilcox, Arizona, USA |
|  | 1840 | ZSCH 1 |  | Malibu, California, USA |
|  | 1878 | SINU |  | Coyne, Oklahoma, USA |
|  | 1879 | SINU |  | Blackburn, Oklahoma, USA |
|  | 1880 | SINU |  | Ralston, Oklahoma, USA |
|  | 18107 | SINU |  | Orr, Oklahoma, USA |
|  | 18109 | SINU |  | Leon, Oklahoma, USA |
|  | 18110 | SINU |  | Asher, Oklahoma, USA |
| *berlandieri* subsp. *berlandieri* var. *zschackei* | 402 | ZSCH 2 |  | San Diego, California, USA |
|  | 423 | ZSCH 2 |  | San Gabriel Mountains, California, USA |
|  | 880 | ZSCH 1 | PI 666294 | Ramah, New Mexico, USA |
|  | 884 | ZSCH 1 |  | Provo, Utah, USA |
|  | 1007 | ZSCH 1 |  | Spring Mountains, Nevada, USA |
|  | 1497 | ZSCH 1 | Ames 32982 | Flagstaff, Arizona, USA |
|  | 1505 | ZSCH 1 |  | Snowville, Utah, USA |
|  | 1516 | ZSCH 1 |  | Cody, Wyoming, USA |
|  | 18102 | ZSCH 1 |  | Sayre, Oklahoma, USA |
|  | 1907 | ZSCH 1 |  | Lakeview, Oregon, USA |
|  | 1909 | ZSCH 1 |  | Lake Almanor, California, USA |
|  | 1913 | ZSCH 1 |  | Montpelier, Idaho, USA |
|  | 19170 | ZSCH 1 |  | Limon, Colorado, USA |

**Supplemental Table S2.** Assembly statistics for the primary contig assembly and the Hi-C scaffolded Canu assemblies.

|  | **Primary Contig Assembly^1^** | **Hi-C scaffolded** |
| --- | --- | --- |
| Number of Contigs | 1170 | 339 |
| Total size of assembly (bp) | 1295427067 | 1295514343 |
| Mean (bp) | 78655 | 3821576 |
| Longest (bp) | 25648010 | 91041477 |
| Shortest (bp) | 1087 | 1087 |
| N50 (bp) | 5248923 | 70051864 |
| L50 | 73 | 9 |
| N count (bp) | 0 | 87300 |
| G +C% | 37.28 | 37.27 |

^1^Primary assembly was polished twice with Illumina data using Arrow followed by a single round of insertion/deletion correction using PILON.

**Supplemental Table S3.** Biosample, alternative names and SRA accessions ID numbers for data deposited in the sequence read archive of GenBank. All samples and sequences are deposited under BioProject PRJNA1026646.

| **BioSample** | **Alternative name** | **NCBI Accession ID** | **SRA ID#** | **Experiment** |
| --- | --- | --- | --- | --- |
| PI433231 | Huauzontle | SAMN37767027 | SRR26349240 | Genome Assembly |
| PI433231 | Huauzontle | SAMN37767027 | SRR26349241 | Genome Assembly |
| PI433231 | Huauzontle | SAMN37767027 | SRR26349239 | Genome Polish |
| PI433231 | Huauzontle | SAMN37767027 | SRR26363924 | HiC data |
| PI433231 | Huauzontle | SAMN37767027 | SRR26349237 | Transcriptome - leaf |
| PI433231 | Huauzontle | SAMN37767027 | SRR26349236 | Transcriptome - seedling |
| PI433231 | Huauzontle | SAMN37767027 | SRR26349238 | Transcriptome - floral |
| PI433231 | Huauzontle | SAMN37767027 | SRR26349235 | Transcriptome - stem |
| Ames 22157 | UDEC-2 | SAMN37754410 | SRR26340536 | ATGC^a^ diversity |
| BYU-1007 | BYU-1007 | SAMN37754412 | SRR26340534 | ATGC diversity |
| BYU-1101 | BYU-1101 | SAMN37754406 | SRR26340540 | ATGC diversity |
| BYU-1312 | BYU-1312 | SAMN37754413 | SRR26340533 | ATGC diversity |
| BYU-1314 | BYU-1314 | SAMN37754414 | SRR26340532 | ATGC diversity |
| BYU-14108 | BYU-14108 | SAMN37754411 | SRR26340535 | ATGC diversity |
| BYU-14111 | BYU-14111 | SAMN37754415 | SRR26340531 | ATGC diversity |
| BYU-14112 | BYU-14112 | SAMN37754416 | SRR26340529 | ATGC diversity |
| BYU-1458 | BYU-1458 | SAMN37754417 | SRR26340528 | ATGC diversity |
| BYU-1465 | BYU-1465 | SAMN37754418 | SRR26340527 | ATGC diversity |
| BYU-1469 | BYU-1469 | SAMN37754419 | SRR26340526 | ATGC diversity |
| BYU-1497 | BYU-1497 | SAMN37754420 | SRR26340525 | ATGC diversity |
| BYU-1505 | BYU-1505 | SAMN37754421 | SRR26340524 | ATGC diversity |
| BYU-1511 | BYU-1511 | SAMN37754422 | SRR26340523 | ATGC diversity |
| BYU-1516 | BYU-1516 | SAMN37754423 | SRR26340522 | ATGC diversity |
| BYU-17127 | BYU-17127 | SAMN37754463 | SRR26340478 | ATGC diversity |
| BYU-17132 | BYU-17132 | SAMN37754424 | SRR26340521 | ATGC diversity |
| BYU-17199 | BYU-17199 | SAMN37754425 | SRR26340520 | ATGC diversity |
| BYU-1770 | BYU-1770 | SAMN37754464 | SRR26340477 | ATGC diversity |
| BYU-1777 | BYU-1777 | SAMN37754465 | SRR26340476 | ATGC diversity |
| BYU-1789 | BYU-1789 | SAMN37754466 | SRR26340474 | ATGC diversity |
| BYU-1804 | BYU-1804 | SAMN37754426 | SRR26340518 | ATGC diversity |
| BYU-18102 | BYU-18102 | SAMN37754427 | SRR26340517 | ATGC diversity |
| BYU-18107 | BYU-18107 | SAMN37754428 | SRR26340516 | ATGC diversity |
| BYU-18109 | BYU-18109 | SAMN37754429 | SRR26340515 | ATGC diversity |
| BYU-18110 | BYU-18110 | SAMN37754430 | SRR26340514 | ATGC diversity |
| BYU-1819 | BYU-1819 | SAMN37754431 | SRR26340513 | ATGC diversity |
| BYU-1821 | BYU-1821 | SAMN37754432 | SRR26340512 | ATGC diversity |
| BYU-1831 | BYU-1831 | SAMN37754433 | SRR26340511 | ATGC diversity |
| BYU-1840 | BYU-1840 | SAMN37754434 | SRR26340510 | ATGC diversity |
| BYU-1856 | BYU-1856 | SAMN37754435 | SRR26340509 | ATGC diversity |
| BYU-1858 | BYU-1858 | SAMN37754436 | SRR26340507 | ATGC diversity |
| BYU-1862 | BYU-1862 | SAMN37754437 | SRR26340506 | ATGC diversity |
| BYU-1863 | BYU-1863 | SAMN37754438 | SRR26340505 | ATGC diversity |
| BYU-1878 | BYU-1878 | SAMN37754439 | SRR26340504 | ATGC diversity |
| BYU-1879 | BYU-1879 | SAMN37754440 | SRR26340503 | ATGC diversity |
| BYU-1880 | BYU-1880 | SAMN37754441 | SRR26340502 | ATGC diversity |
| BYU-1891 | BYU-1891 | SAMN37754442 | SRR26340501 | ATGC diversity |
| BYU-1896 | BYU-1896 | SAMN37754443 | SRR26340500 | ATGC diversity |
| BYU-1897 | BYU-1897 | SAMN37754444 | SRR26340499 | ATGC diversity |
| BYU-1904 | BYU-1904 | SAMN37754467 | SRR26340473 | ATGC diversity |
| BYU-1907 | BYU-1907 | SAMN37754445 | SRR26340498 | ATGC diversity |
| BYU-1909 | BYU-1909 | SAMN37754446 | SRR26340496 | ATGC diversity |
| BYU-19127 | BYU-19127 | SAMN37754447 | SRR26340495 | ATGC diversity |
| BYU-1913 | BYU-1913 | SAMN37754448 | SRR26340494 | ATGC diversity |
| BYU-19146 | BYU-19146 | SAMN37754449 | SRR26340493 | ATGC diversity |
| BYU-19170 | BYU-19170 | SAMN37754450 | SRR26340492 | ATGC diversity |
| BYU-19239 | BYU-19239 | SAMN37754451 | SRR26340491 | ATGC diversity |
| BYU-19259 | BYU-19259 | SAMN37754452 | SRR26340490 | ATGC diversity |
| BYU-19302 | BYU-19302 | SAMN37754453 | SRR26340489 | ATGC diversity |
| BYU-19306 | BYU-19306 | SAMN37754454 | SRR26340488 | ATGC diversity |
| BYU-1965 | BYU-1965 | SAMN37754455 | SRR26340487 | ATGC diversity |
| BYU-2004 | BYU-2004 | SAMN37754456 | SRR26340485 | ATGC diversity |
| BYU-402 | BYU-402 | SAMN37754457 | SRR26340484 | ATGC diversity |
| BYU-423 | BYU-423 | SAMN37754458 | SRR26340483 | ATGC diversity |
| BYU-566 | BYU-566 | SAMN37754400 | SRR26340508 | ATGC diversity |
| BYU-803 | BYU-803 | SAMN37754459 | SRR26340482 | ATGC diversity |
| BYU-880 | BYU-880 | SAMN37754460 | SRR26340481 | ATGC diversity |
| BYU-884 | BYU-884 | SAMN37754461 | SRR26340480 | ATGC diversity |
| BYU-937 | BYU-937 | SAMN37754462 | SRR26340479 | ATGC diversity |
| BYU-1439 | Cherry Vanilla | SAMN37754407 | SRR26340539 | ATGC diversity |
| BYU-1650 | Chucapaca | SAMN37754408 | SRR26340538 | ATGC diversity |
| BYU-1613 | CICA-17 | SAMN37754402 | SRR26340486 | ATGC diversity |
| BYU-1624 | G-205-95-DK | SAMN37754405 | SRR26340453 | ATGC diversity |
| BYU-17176 | J1 | SAMN37754468 | SRR26340472 | ATGC diversity |
| BYU-19286 | J2 | SAMN37754478 | SRR26340461 | ATGC diversity |
| BYU-1663 | J3 | SAMN37754479 | SRR26340460 | ATGC diversity |
| BYU-19287 | J4 | SAMN37754480 | SRR26340459 | ATGC diversity |
| BYU-1447 | J5 | SAMN37754481 | SRR26340458 | ATGC diversity |
| BYU-1647 | J6 | SAMN37754482 | SRR26340457 | ATGC diversity |
| BYU-1483 | J7 | SAMN37754483 | SRR26340456 | ATGC diversity |
| BYU-1664 | J8 | SAMN37754484 | SRR26340455 | ATGC diversity |
| BYU-1484 | J9 | SAMN37754485 | SRR26340454 | ATGC diversity |
| BYU-1669 | J10 | SAMN37754469 | SRR26340471 | ATGC diversity |
| BYU-1662 | J11 | SAMN37754470 | SRR26340470 | ATGC diversity |
| BYU-567 | J12 | SAMN37754471 | SRR26340469 | ATGC diversity |
| BYU-1668 | J13 | SAMN37754472 | SRR26340468 | ATGC diversity |
| BYU-1666 | J14 | SAMN37754473 | SRR26340467 | ATGC diversity |
| BYU-668 | J15 | SAMN37754474 | SRR26340466 | ATGC diversity |
| BYU-1486 | J16 | SAMN37754475 | SRR26340465 | ATGC diversity |
| BYU-1485 | J17 | SAMN37754476 | SRR26340463 | ATGC diversity |
| BYU-19284 | J18 | SAMN37754477 | SRR26340462 | ATGC diversity |
| BYU-2044 | KU-2 | SAMN37754409 | SRR26340537 | ATGC diversity |
| LP | Kurmi | SAMN37754401 | SRR26340497 | ATGC diversity |
| BYU-1648 | O654 | SAMN37754396 | SRR26340542 | ATGC diversity |
| BYU-1615 | Ollague | SAMN37754397 | SRR26340541 | ATGC diversity |
| BYU-1202 | Pasankalla | SAMN37754399 | SRR26340519 | ATGC diversity |
| BYU-1633 | Real-1 | SAMN37754398 | SRR26340530 | ATGC diversity |
| BYU-947 | Regalona | SAMN37754403 | SRR26340475 | ATGC diversity |
| BYU-1842 | Sacedo-INIA | SAMN37754404 | SRR26340464 | ATGC diversity |
| BYU-1101_parent | Parent | SAMN37795111 | SRR26366901 | Extranuclear inheritance |
| BYU-937_parent | Parent | SAMN37795112 | SRR26366900 | Extranuclear inheritance |
| Real-1_parent | Parent | SAMN37795113 | SRR26366899 | Extranuclear inheritance |
| RealX1101 | F1 | SAMN37795115 | SRR26366897 | Extranuclear inheritance |
| RealX937 | F1 | SAMN37795114 | SRR26366898 | Extranuclear inheritance |
| R11-1 | RealXBYU1101_F2 | SAMN37795101 | SRR26366912 | Linkage mapping |
| R11-10 | RealXBYU1101_F2 | SAMN37795060 | SRR26366992 | Linkage mapping |
| R11-101 | RealXBYU1101_F2 | SAMN37795030 | SRR26366864 | Linkage mapping |
| R11-11 | RealXBYU1101_F2 | SAMN37795028 | SRR26366866 | Linkage mapping |
| R11-12 | RealXBYU1101_F2 | SAMN37795043 | SRR26367010 | Linkage mapping |
| R11-120 | RealXBYU1101_F2 | SAMN37795108 | SRR26366905 | Linkage mapping |
| R11-13 | RealXBYU1101_F2 | SAMN37795054 | SRR26366998 | Linkage mapping |
| R11-14 | RealXBYU1101_F2 | SAMN37795040 | SRR26367014 | Linkage mapping |
| R11-15 | RealXBYU1101_F2 | SAMN37795094 | SRR26366920 | Linkage mapping |
| R11-16 | RealXBYU1101_F2 | SAMN37795039 | SRR26367015 | Linkage mapping |
| R11-17 | RealXBYU1101_F2 | SAMN37795049 | SRR26367004 | Linkage mapping |
| R11-18 | RealXBYU1101_F2 | SAMN37795034 | SRR26367020 | Linkage mapping |
| R11-19 | RealXBYU1101_F2 | SAMN37795050 | SRR26367003 | Linkage mapping |
| R11-2 | RealXBYU1101_F2 | SAMN37795106 | SRR26366907 | Linkage mapping |
| R11-20 | RealXBYU1101_F2 | SAMN37795055 | SRR26366997 | Linkage mapping |
| R11-21 | RealXBYU1101_F2 | SAMN37795082 | SRR26366805 | Linkage mapping |
| R11-22 | RealXBYU1101_F2 | SAMN37795027 | SRR26366867 | Linkage mapping |
| R11-23 | RealXBYU1101_F2 | SAMN37795036 | SRR26367018 | Linkage mapping |
| R11-24 | RealXBYU1101_F2 | SAMN37795076 | SRR26366812 | Linkage mapping |
| R11-25 | RealXBYU1101_F2 | SAMN37795029 | SRR26366865 | Linkage mapping |
| R11-26 | RealXBYU1101_F2 | SAMN37795033 | SRR26367021 | Linkage mapping |
| R11-27 | RealXBYU1101_F2 | SAMN37795100 | SRR26366914 | Linkage mapping |
| R11-28 | RealXBYU1101_F2 | SAMN37795105 | SRR26366908 | Linkage mapping |
| R11-29 | RealXBYU1101_F2 | SAMN37795067 | SRR26366823 | Linkage mapping |
| R11-3 | RealXBYU1101_F2 | SAMN37795069 | SRR26366821 | Linkage mapping |
| R11-30 | RealXBYU1101_F2 | SAMN37795059 | SRR26366993 | Linkage mapping |
| R11-31 | RealXBYU1101_F2 | SAMN37795042 | SRR26367011 | Linkage mapping |
| R11-32 | RealXBYU1101_F2 | SAMN37795104 | SRR26366909 | Linkage mapping |
| R11-33 | RealXBYU1101_F2 | SAMN37795025 | SRR26366869 | Linkage mapping |
| R11-34 | RealXBYU1101_F2 | SAMN37795053 | SRR26366999 | Linkage mapping |
| R11-35 | RealXBYU1101_F2 | SAMN37795079 | SRR26366809 | Linkage mapping |
| R11-36 | RealXBYU1101_F2 | SAMN37795071 | SRR26366817 | Linkage mapping |
| R11-37 | RealXBYU1101_F2 | SAMN37795035 | SRR26367019 | Linkage mapping |
| R11-38 | RealXBYU1101_F2 | SAMN37795075 | SRR26366813 | Linkage mapping |
| R11-39 | RealXBYU1101_F2 | SAMN37795051 | SRR26367001 | Linkage mapping |
| R11-4 | RealXBYU1101_F2 | SAMN37795072 | SRR26366816 | Linkage mapping |
| R11-40 | RealXBYU1101_F2 | SAMN37795052 | SRR26367000 | Linkage mapping |
| R11-41 | RealXBYU1101_F2 | SAMN37795065 | SRR26366825 | Linkage mapping |
| R11-42 | RealXBYU1101_F2 | SAMN37795081 | SRR26366806 | Linkage mapping |
| R11-43 | RealXBYU1101_F2 | SAMN37795026 | SRR26366868 | Linkage mapping |
| R11-44 | RealXBYU1101_F2 | SAMN37795077 | SRR26366811 | Linkage mapping |
| R11-45 | RealXBYU1101_F2 | SAMN37795110 | SRR26366903 | Linkage mapping |
| R11-46 | RealXBYU1101_F2 | SAMN37795057 | SRR26366995 | Linkage mapping |
| R11-47 | RealXBYU1101_F2 | SAMN37795038 | SRR26367016 | Linkage mapping |
| R11-48 | RealXBYU1101_F2 | SAMN37795063 | SRR26366827 | Linkage mapping |
| R11-49 | RealXBYU1101_F2 | SAMN37795089 | SRR26366926 | Linkage mapping |
| R11-5 | RealXBYU1101_F2 | SAMN37795068 | SRR26366822 | Linkage mapping |
| R11-50 | RealXBYU1101_F2 | SAMN37795041 | SRR26367012 | Linkage mapping |
| R11-51 | RealXBYU1101_F2 | SAMN37795062 | SRR26366828 | Linkage mapping |
| R11-52 | RealXBYU1101_F2 | SAMN37795056 | SRR26366996 | Linkage mapping |
| R11-53 | RealXBYU1101_F2 | SAMN37795070 | SRR26366820 | Linkage mapping |
| R11-54 | RealXBYU1101_F2 | SAMN37795090 | SRR26366925 | Linkage mapping |
| R11-55 | RealXBYU1101_F2 | SAMN37795091 | SRR26366923 | Linkage mapping |
| R11-56 | RealXBYU1101_F2 | SAMN37795066 | SRR26366824 | Linkage mapping |
| R11-57 | RealXBYU1101_F2 | SAMN37795078 | SRR26366810 | Linkage mapping |
| R11-58 | RealXBYU1101_F2 | SAMN37795045 | SRR26367008 | Linkage mapping |
| R11-59 | RealXBYU1101_F2 | SAMN37795093 | SRR26366921 | Linkage mapping |
| R11-6 | RealXBYU1101_F2 | SAMN37795058 | SRR26366994 | Linkage mapping |
| R11-60 | RealXBYU1101_F2 | SAMN37795096 | SRR26366918 | Linkage mapping |
| R11-61 | RealXBYU1101_F2 | SAMN37795085 | SRR26366802 | Linkage mapping |
| R11-62 | RealXBYU1101_F2 | SAMN37795083 | SRR26366804 | Linkage mapping |
| R11-63 | RealXBYU1101_F2 | SAMN37795086 | SRR26366801 | Linkage mapping |
| R11-64 | RealXBYU1101_F2 | SAMN37795107 | SRR26366906 | Linkage mapping |
| R11-65 | RealXBYU1101_F2 | SAMN37795080 | SRR26366808 | Linkage mapping |
| R11-66 | RealXBYU1101_F2 | SAMN37795064 | SRR26366826 | Linkage mapping |
| R11-67 | RealXBYU1101_F2 | SAMN37795092 | SRR26366922 | Linkage mapping |
| R11-68 | RealXBYU1101_F2 | SAMN37795074 | SRR26366814 | Linkage mapping |
| R11-69 | RealXBYU1101_F2 | SAMN37795073 | SRR26366815 | Linkage mapping |
| R11-7 | RealXBYU1101_F2 | SAMN37795044 | SRR26367009 | Linkage mapping |
| R11-70 | RealXBYU1101_F2 | SAMN37795098 | SRR26366916 | Linkage mapping |
| R11-71 | RealXBYU1101_F2 | SAMN37795048 | SRR26367005 | Linkage mapping |
| R11-76 | RealXBYU1101_F2 | SAMN37795031 | SRR26367023 | Linkage mapping |
| R11-78 | RealXBYU1101_F2 | SAMN37795084 | SRR26366803 | Linkage mapping |
| R11-79 | RealXBYU1101_F2 | SAMN37795102 | SRR26366911 | Linkage mapping |
| R11-8 | RealXBYU1101_F2 | SAMN37795061 | SRR26366829 | Linkage mapping |
| R11-80 | RealXBYU1101_F2 | SAMN37795037 | SRR26367017 | Linkage mapping |
| R11-81 | RealXBYU1101_F2 | SAMN37795095 | SRR26366919 | Linkage mapping |
| R11-82 | RealXBYU1101_F2 | SAMN37795088 | SRR26366798 | Linkage mapping |
| R11-83 | RealXBYU1101_F2 | SAMN37795099 | SRR26366915 | Linkage mapping |
| R11-84 | RealXBYU1101_F2 | SAMN37795047 | SRR26367006 | Linkage mapping |
| R11-86 | RealXBYU1101_F2 | SAMN37795097 | SRR26366917 | Linkage mapping |
| R11-9 | RealXBYU1101_F2 | SAMN37795032 | SRR26367022 | Linkage mapping |
| R11-90 | RealXBYU1101_F2 | SAMN37795103 | SRR26366910 | Linkage mapping |
| R11-93 | RealXBYU1101_F2 | SAMN37795046 | SRR26367007 | Linkage mapping |
| R11-97 | RealXBYU1101_F2 | SAMN37795109 | SRR26366904 | Linkage mapping |
| R11-99 | RealXBYU1101_F2 | SAMN37795087 | SRR26366800 | Linkage mapping |
| R9-1 | RealXBYU937_F2 | SAMN37794871 | SRR26367027 | Linkage mapping |
| R9-10 | RealXBYU937_F2 | SAMN37794872 | SRR26367026 | Linkage mapping |
| R9-100 | RealXBYU937_F2 | SAMN37794873 | SRR26366819 | Linkage mapping |
| R9-102 | RealXBYU937_F2 | SAMN37794874 | SRR26366859 | Linkage mapping |
| R9-103 | RealXBYU937_F2 | SAMN37794875 | SRR26366848 | Linkage mapping |
| R9-104 | RealXBYU937_F2 | SAMN37794876 | SRR26366837 | Linkage mapping |
| R9-106 | RealXBYU937_F2 | SAMN37794877 | SRR26366987 | Linkage mapping |
| R9-108 | RealXBYU937_F2 | SAMN37794878 | SRR26366976 | Linkage mapping |
| R9-109 | RealXBYU937_F2 | SAMN37794879 | SRR26366965 | Linkage mapping |
| R9-11 | RealXBYU937_F2 | SAMN37794880 | SRR26366793 | Linkage mapping |
| R9-110 | RealXBYU937_F2 | SAMN37794881 | SRR26367025 | Linkage mapping |
| R9-112 | RealXBYU937_F2 | SAMN37794882 | SRR26366950 | Linkage mapping |
| R9-113 | RealXBYU937_F2 | SAMN37794883 | SRR26366939 | Linkage mapping |
| R9-114 | RealXBYU937_F2 | SAMN37794884 | SRR26366928 | Linkage mapping |
| R9-115 | RealXBYU937_F2 | SAMN37794885 | SRR26366885 | Linkage mapping |
| R9-116 | RealXBYU937_F2 | SAMN37794886 | SRR26366874 | Linkage mapping |
| R9-117 | RealXBYU937_F2 | SAMN37794887 | SRR26366863 | Linkage mapping |
| R9-118 | RealXBYU937_F2 | SAMN37794888 | SRR26367013 | Linkage mapping |
| R9-119 | RealXBYU937_F2 | SAMN37794889 | SRR26367002 | Linkage mapping |
| R9-12 | RealXBYU937_F2 | SAMN37794890 | SRR26366830 | Linkage mapping |
| R9-120 | RealXBYU937_F2 | SAMN37794891 | SRR26366818 | Linkage mapping |
| R9-121 | RealXBYU937_F2 | SAMN37794892 | SRR26366807 | Linkage mapping |
| R9-122 | RealXBYU937_F2 | SAMN37794893 | SRR26366924 | Linkage mapping |
| R9-124 | RealXBYU937_F2 | SAMN37794894 | SRR26366913 | Linkage mapping |
| R9-125 | RealXBYU937_F2 | SAMN37794895 | SRR26366902 | Linkage mapping |
| R9-126 | RealXBYU937_F2 | SAMN37794896 | SRR26366896 | Linkage mapping |
| R9-128 | RealXBYU937_F2 | SAMN37794897 | SRR26366895 | Linkage mapping |
| R9-129 | RealXBYU937_F2 | SAMN37794898 | SRR26366862 | Linkage mapping |
| R9-13 | RealXBYU937_F2 | SAMN37794899 | SRR26366861 | Linkage mapping |
| R9-130 | RealXBYU937_F2 | SAMN37794900 | SRR26366860 | Linkage mapping |
| R9-131 | RealXBYU937_F2 | SAMN37794901 | SRR26366858 | Linkage mapping |
| R9-132 | RealXBYU937_F2 | SAMN37794902 | SRR26366857 | Linkage mapping |
| R9-133 | RealXBYU937_F2 | SAMN37794903 | SRR26366856 | Linkage mapping |
| R9-134 | RealXBYU937_F2 | SAMN37794904 | SRR26366855 | Linkage mapping |
| R9-136 | RealXBYU937_F2 | SAMN37794905 | SRR26366854 | Linkage mapping |
| R9-137 | RealXBYU937_F2 | SAMN37794906 | SRR26366853 | Linkage mapping |
| R9-138 | RealXBYU937_F2 | SAMN37794907 | SRR26366852 | Linkage mapping |
| R9-139 | RealXBYU937_F2 | SAMN37794908 | SRR26366851 | Linkage mapping |
| R9-14 | RealXBYU937_F2 | SAMN37794909 | SRR26366850 | Linkage mapping |
| R9-140 | RealXBYU937_F2 | SAMN37794910 | SRR26366849 | Linkage mapping |
| R9-141 | RealXBYU937_F2 | SAMN37794911 | SRR26366847 | Linkage mapping |
| R9-142 | RealXBYU937_F2 | SAMN37794912 | SRR26366846 | Linkage mapping |
| R9-143 | RealXBYU937_F2 | SAMN37794913 | SRR26366845 | Linkage mapping |
| R9-144 | RealXBYU937_F2 | SAMN37794914 | SRR26366844 | Linkage mapping |
| R9-145 | RealXBYU937_F2 | SAMN37794915 | SRR26366843 | Linkage mapping |
| R9-146 | RealXBYU937_F2 | SAMN37794916 | SRR26366842 | Linkage mapping |
| R9-147 | RealXBYU937_F2 | SAMN37794917 | SRR26366841 | Linkage mapping |
| R9-148 | RealXBYU937_F2 | SAMN37794918 | SRR26366840 | Linkage mapping |
| R9-151 | RealXBYU937_F2 | SAMN37794919 | SRR26366839 | Linkage mapping |
| R9-152 | RealXBYU937_F2 | SAMN37794920 | SRR26366838 | Linkage mapping |
| R9-155 | RealXBYU937_F2 | SAMN37794921 | SRR26366836 | Linkage mapping |
| R9-156 | RealXBYU937_F2 | SAMN37794922 | SRR26366835 | Linkage mapping |
| R9-157 | RealXBYU937_F2 | SAMN37794923 | SRR26366834 | Linkage mapping |
| R9-158 | RealXBYU937_F2 | SAMN37794924 | SRR26366833 | Linkage mapping |
| R9-159 | RealXBYU937_F2 | SAMN37794925 | SRR26366832 | Linkage mapping |
| R9-16 | RealXBYU937_F2 | SAMN37794926 | SRR26366831 | Linkage mapping |
| R9-160 | RealXBYU937_F2 | SAMN37794927 | SRR26366991 | Linkage mapping |
| R9-161 | RealXBYU937_F2 | SAMN37794928 | SRR26366990 | Linkage mapping |
| R9-162 | RealXBYU937_F2 | SAMN37794929 | SRR26366989 | Linkage mapping |
| R9-163 | RealXBYU937_F2 | SAMN37794930 | SRR26366988 | Linkage mapping |
| R9-164 | RealXBYU937_F2 | SAMN37794931 | SRR26366986 | Linkage mapping |
| R9-165 | RealXBYU937_F2 | SAMN37794932 | SRR26366985 | Linkage mapping |
| R9-166 | RealXBYU937_F2 | SAMN37794933 | SRR26366984 | Linkage mapping |
| R9-167 | RealXBYU937_F2 | SAMN37794934 | SRR26366983 | Linkage mapping |
| R9-168 | RealXBYU937_F2 | SAMN37794935 | SRR26366982 | Linkage mapping |
| R9-169 | RealXBYU937_F2 | SAMN37794936 | SRR26366981 | Linkage mapping |
| R9-17 | RealXBYU937_F2 | SAMN37794937 | SRR26366980 | Linkage mapping |
| R9-170 | RealXBYU937_F2 | SAMN37794938 | SRR26366979 | Linkage mapping |
| R9-171 | RealXBYU937_F2 | SAMN37794939 | SRR26366978 | Linkage mapping |
| R9-172 | RealXBYU937_F2 | SAMN37794940 | SRR26366977 | Linkage mapping |
| R9-174 | RealXBYU937_F2 | SAMN37794941 | SRR26366975 | Linkage mapping |
| R9-176 | RealXBYU937_F2 | SAMN37794942 | SRR26366974 | Linkage mapping |
| R9-177 | RealXBYU937_F2 | SAMN37794943 | SRR26366973 | Linkage mapping |
| R9-179 | RealXBYU937_F2 | SAMN37794944 | SRR26366972 | Linkage mapping |
| R9-18 | RealXBYU937_F2 | SAMN37794945 | SRR26366971 | Linkage mapping |
| R9-181 | RealXBYU937_F2 | SAMN37794946 | SRR26366970 | Linkage mapping |
| R9-182 | RealXBYU937_F2 | SAMN37794947 | SRR26366969 | Linkage mapping |
| R9-183 | RealXBYU937_F2 | SAMN37794948 | SRR26366968 | Linkage mapping |
| R9-184 | RealXBYU937_F2 | SAMN37794949 | SRR26366967 | Linkage mapping |
| R9-185 | RealXBYU937_F2 | SAMN37794950 | SRR26366966 | Linkage mapping |
| R9-19 | RealXBYU937_F2 | SAMN37794951 | SRR26366964 | Linkage mapping |
| R9-2 | RealXBYU937_F2 | SAMN37794952 | SRR26366963 | Linkage mapping |
| R9-20 | RealXBYU937_F2 | SAMN37794953 | SRR26366962 | Linkage mapping |
| R9-21 | RealXBYU937_F2 | SAMN37794954 | SRR26366961 | Linkage mapping |
| R9-22 | RealXBYU937_F2 | SAMN37794955 | SRR26366960 | Linkage mapping |
| R9-24 | RealXBYU937_F2 | SAMN37794956 | SRR26366799 | Linkage mapping |
| R9-25 | RealXBYU937_F2 | SAMN37794957 | SRR26366797 | Linkage mapping |
| R9-26 | RealXBYU937_F2 | SAMN37794958 | SRR26366796 | Linkage mapping |
| R9-27 | RealXBYU937_F2 | SAMN37794959 | SRR26366795 | Linkage mapping |
| R9-28 | RealXBYU937_F2 | SAMN37794960 | SRR26366794 | Linkage mapping |
| R9-29 | RealXBYU937_F2 | SAMN37794961 | SRR26366792 | Linkage mapping |
| R9-3 | RealXBYU937_F2 | SAMN37794962 | SRR26366791 | Linkage mapping |
| R9-30 | RealXBYU937_F2 | SAMN37794963 | SRR26366790 | Linkage mapping |
| R9-31 | RealXBYU937_F2 | SAMN37794964 | SRR26366789 | Linkage mapping |
| R9-32 | RealXBYU937_F2 | SAMN37794965 | SRR26366788 | Linkage mapping |
| R9-33 | RealXBYU937_F2 | SAMN37794966 | SRR26366787 | Linkage mapping |
| R9-34 | RealXBYU937_F2 | SAMN37794967 | SRR26366786 | Linkage mapping |
| R9-35 | RealXBYU937_F2 | SAMN37794968 | SRR26366785 | Linkage mapping |
| R9-37 | RealXBYU937_F2 | SAMN37794969 | SRR26366784 | Linkage mapping |
| R9-39 | RealXBYU937_F2 | SAMN37794970 | SRR26366783 | Linkage mapping |
| R9-4 | RealXBYU937_F2 | SAMN37794971 | SRR26367024 | Linkage mapping |
| R9-41 | RealXBYU937_F2 | SAMN37794972 | SRR26366959 | Linkage mapping |
| R9-42 | RealXBYU937_F2 | SAMN37794973 | SRR26366958 | Linkage mapping |
| R9-43 | RealXBYU937_F2 | SAMN37794974 | SRR26366957 | Linkage mapping |
| R9-44 | RealXBYU937_F2 | SAMN37794975 | SRR26366956 | Linkage mapping |
| R9-45 | RealXBYU937_F2 | SAMN37794976 | SRR26366955 | Linkage mapping |
| R9-46 | RealXBYU937_F2 | SAMN37794977 | SRR26366954 | Linkage mapping |
| R9-47 | RealXBYU937_F2 | SAMN37794978 | SRR26366953 | Linkage mapping |
| R9-48 | RealXBYU937_F2 | SAMN37794979 | SRR26366952 | Linkage mapping |
| R9-49 | RealXBYU937_F2 | SAMN37794980 | SRR26366951 | Linkage mapping |
| R9-5 | RealXBYU937_F2 | SAMN37794981 | SRR26366949 | Linkage mapping |
| R9-50 | RealXBYU937_F2 | SAMN37794982 | SRR26366948 | Linkage mapping |
| R9-52 | RealXBYU937_F2 | SAMN37794983 | SRR26366947 | Linkage mapping |
| R9-54 | RealXBYU937_F2 | SAMN37794984 | SRR26366946 | Linkage mapping |
| R9-55 | RealXBYU937_F2 | SAMN37794985 | SRR26366945 | Linkage mapping |
| R9-56 | RealXBYU937_F2 | SAMN37794986 | SRR26366944 | Linkage mapping |
| R9-57 | RealXBYU937_F2 | SAMN37794987 | SRR26366943 | Linkage mapping |
| R9-58 | RealXBYU937_F2 | SAMN37794988 | SRR26366942 | Linkage mapping |
| R9-59 | RealXBYU937_F2 | SAMN37794989 | SRR26366941 | Linkage mapping |
| R9-60 | RealXBYU937_F2 | SAMN37794990 | SRR26366940 | Linkage mapping |
| R9-61 | RealXBYU937_F2 | SAMN37794991 | SRR26366938 | Linkage mapping |
| R9-62 | RealXBYU937_F2 | SAMN37794992 | SRR26366937 | Linkage mapping |
| R9-63 | RealXBYU937_F2 | SAMN37794993 | SRR26366936 | Linkage mapping |
| R9-65 | RealXBYU937_F2 | SAMN37794994 | SRR26366935 | Linkage mapping |
| R9-66 | RealXBYU937_F2 | SAMN37794995 | SRR26366934 | Linkage mapping |
| R9-67 | RealXBYU937_F2 | SAMN37794996 | SRR26366933 | Linkage mapping |
| R9-68 | RealXBYU937_F2 | SAMN37794997 | SRR26366932 | Linkage mapping |
| R9-7 | RealXBYU937_F2 | SAMN37794998 | SRR26366931 | Linkage mapping |
| R9-70 | RealXBYU937_F2 | SAMN37794999 | SRR26366930 | Linkage mapping |
| R9-71 | RealXBYU937_F2 | SAMN37795000 | SRR26366929 | Linkage mapping |
| R9-74 | RealXBYU937_F2 | SAMN37795001 | SRR26366927 | Linkage mapping |
| R9-75 | RealXBYU937_F2 | SAMN37795002 | SRR26366894 | Linkage mapping |
| R9-76 | RealXBYU937_F2 | SAMN37795003 | SRR26366893 | Linkage mapping |
| R9-77 | RealXBYU937_F2 | SAMN37795004 | SRR26366892 | Linkage mapping |
| R9-78 | RealXBYU937_F2 | SAMN37795005 | SRR26366891 | Linkage mapping |
| R9-79 | RealXBYU937_F2 | SAMN37795006 | SRR26366890 | Linkage mapping |
| R9-8 | RealXBYU937_F2 | SAMN37795007 | SRR26366889 | Linkage mapping |
| R9-80 | RealXBYU937_F2 | SAMN37795008 | SRR26366888 | Linkage mapping |
| R9-81 | RealXBYU937_F2 | SAMN37795009 | SRR26366887 | Linkage mapping |
| R9-82 | RealXBYU937_F2 | SAMN37795010 | SRR26366886 | Linkage mapping |
| R9-83 | RealXBYU937_F2 | SAMN37795011 | SRR26366884 | Linkage mapping |
| R9-84 | RealXBYU937_F2 | SAMN37795012 | SRR26366883 | Linkage mapping |
| R9-85 | RealXBYU937_F2 | SAMN37795013 | SRR26366882 | Linkage mapping |
| R9-86 | RealXBYU937_F2 | SAMN37795014 | SRR26366881 | Linkage mapping |
| R9-88 | RealXBYU937_F2 | SAMN37795015 | SRR26366880 | Linkage mapping |
| R9-89 | RealXBYU937_F2 | SAMN37795016 | SRR26366879 | Linkage mapping |
| R9-9 | RealXBYU937_F2 | SAMN37795017 | SRR26366878 | Linkage mapping |
| R9-90 | RealXBYU937_F2 | SAMN37795018 | SRR26366877 | Linkage mapping |
| R9-91 | RealXBYU937_F2 | SAMN37795019 | SRR26366876 | Linkage mapping |
| R9-92 | RealXBYU937_F2 | SAMN37795020 | SRR26366875 | Linkage mapping |
| R9-95 | RealXBYU937_F2 | SAMN37795021 | SRR26366873 | Linkage mapping |
| R9-96 | RealXBYU937_F2 | SAMN37795022 | SRR26366872 | Linkage mapping |
| R9-97 | RealXBYU937_F2 | SAMN37795023 | SRR26366871 | Linkage mapping |
| R9-99 | RealXBYU937_F2 | SAMN37795024 | SRR26366870 | Linkage mapping |

^a^ATGC=Allotetraploid goosefoot complex.
